# Sexually dimorphic roles for the type 2 diabetes-associated *C2cd4b* gene in murine glucose homeostasis

**DOI:** 10.1007/s00125-020-05350-x

**Published:** 2021-01-25

**Authors:** S. Neda Mousavy Gharavy, Bryn M. Owen, Steven J. Millership, Pauline Chabosseau, Grazia Pizza, Aida Martinez-Sanchez, Emirhan Tasoez, Eleni Georgiadou, Ming Hu, Nicholas H. F. Fine, David A. Jacobson, Matthew T. Dickerson, Olof Idevall-Hagren, Alex Montoya, Holger Kramer, Zenobia Mehta, Dominic J. Withers, Nikolay Ninov, Paul J. Gadue, Fabian L. Cardenas-Diaz, Céline Cruciani-Guglielmacci, Christophe Magnan, Mark Ibberson, Isabelle Leclerc, Marianne Voz, Guy A. Rutter

**Affiliations:** 1 Section of Cell Biology and Functional Genomics, Department of Metabolism, Digestion and Reproduction, Imperial College London, Hammersmith Hospital, London, UK; 2 Section of Investigative Medicine, Department of Metabolism, Digestion and Reproduction, Imperial College London, Hammersmith Hospital, London, UK; 3 MRC London Institute of Medical Sciences, Imperial College London, Hammersmith Campus, London, UK; 4 Institute of Clinical Sciences, Faculty of Medicine, Imperial College London, London, UK; 5 DFG-Center for Regenerative Therapies, Technische Universität Dresden, Dresden, Germany; 6 Department of Molecular Physiology and Biophysics Vanderbilt University, Nashville, TN USA; 7 Department of Medical Cell Biology, Uppsala University, Uppsala, Sweden; 8 Children’s Hospital of Philadelphia, CTRB, Philadelphia, PA USA; 9 Regulation of Glycemia by Central Nervous System, BFA, UMR 8251, CNRS Université de Paris, Paris, France; 10 Vital-IT Group, SIB Swiss Institute of Bioinformatics, Lausanne, Switzerland; 11 Laboratory of Zebrafish Development and Disease Models, University of Liège (ULg), Liège, Belgium; 12 Lee Kong Chian School of Medicine, Nanyang Technological University, Singapore, Singapore

**Keywords:** C2CD4A/B, Follicle-stimulating hormone, Genome-wide association studies, Glucose homeostasis, Type 2 diabetes

## Abstract

**Aims/hypothesis:**

Variants close to the *VPS13C*/*C2CD4A/C2CD4B* locus are associated with altered risk of type 2 diabetes in genome-wide association studies. While previous functional work has suggested roles for *VPS13C* and *C2CD4A* in disease development, none has explored the role of *C2CD4B*.

**Methods:**

CRISPR/Cas9-induced global *C2cd4b*-knockout mice and zebrafish larvae with *c2cd4a* deletion were used to study the role of this gene in glucose homeostasis. C2 calcium dependent domain containing protein (C2CD)4A and C2CD4B constructs tagged with FLAG or green fluorescent protein were generated to investigate subcellular dynamics using confocal or near-field microscopy and to identify interacting partners by mass spectrometry.

**Results:**

Systemic inactivation of *C2cd4b* in mice led to marked, but highly sexually dimorphic changes in body weight and glucose homeostasis. Female *C2cd4b* mice displayed unchanged body weight compared with control littermates, but abnormal glucose tolerance (AUC, *p* = 0.01) and defective in vivo, but not in vitro, insulin secretion (*p* = 0.02). This was associated with a marked decrease in follicle-stimulating hormone levels as compared with wild-type (WT) littermates (*p* = 0.003). In sharp contrast, male *C2cd4b* null mice displayed essentially normal glucose tolerance but an increase in body weight (*p* < 0.001) and fasting blood glucose (*p* = 0.003) after maintenance on a high-fat and -sucrose diet vs WT littermates. No metabolic disturbances were observed after global inactivation of *C2cd4a* in mice, or in pancreatic beta cell function at larval stages in *C2cd4a* null zebrafish. Fasting blood glucose levels were also unaltered in adult *C2cd4a*-null fish. C2CD4B and C2CD4A were partially localised to the plasma membrane, with the latter under the control of intracellular Ca^2+^. Binding partners for both included secretory-granule-localised PTPRN2/phogrin.

**Conclusions/interpretation:**

Our studies suggest that *C2cd4b* may act centrally in the pituitary to influence sex-dependent circuits that control pancreatic beta cell function and glucose tolerance in rodents. However, the absence of sexual dimorphism in the impact of diabetes risk variants argues for additional roles for *C2CD4A* or *VPS13C* in the control of glucose homeostasis in humans.

**Data availability:**

The datasets generated and/or analysed during the current study are available in the Biorxiv repository (www.biorxiv.org/content/10.1101/2020.05.18.099200v1). RNA-Seq (GSE152576) and proteomics (PXD021597) data have been deposited to GEO (www.ncbi.nlm.nih.gov/geo/query/acc.cgi?acc=GSE152576) and ProteomeXchange (www.ebi.ac.uk/pride/archive/projects/PXD021597) repositories, respectively.

**Graphical abstract:**

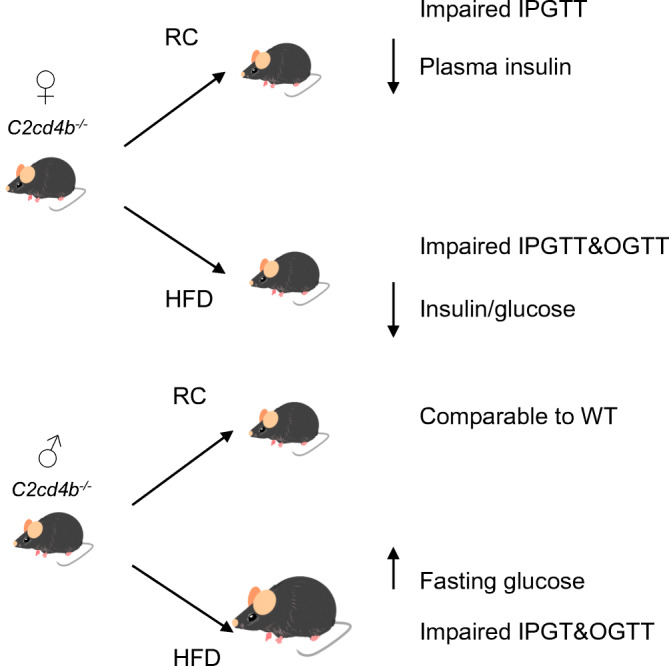

**Supplementary Information:**

The online version contains peer-reviewed but unedited supplementary material available at 10.1007/s00125-020-05350-x.

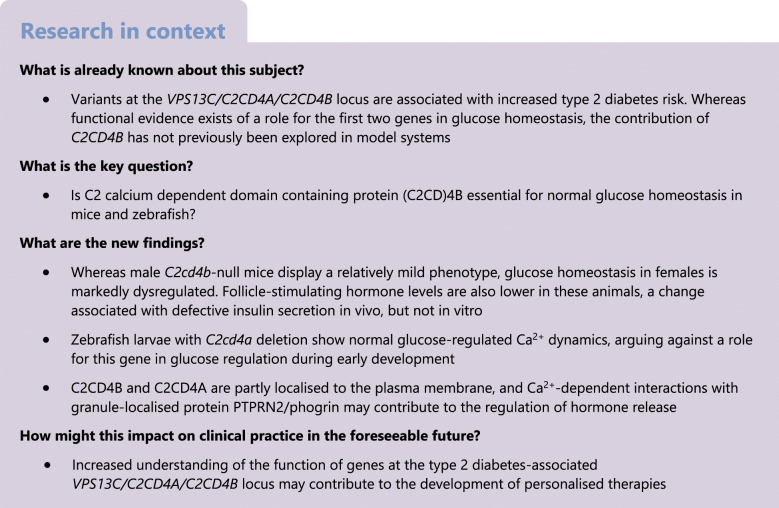



## Introduction

Type 2 diabetes risk is the product of both environmental and genetic factors. More than 200 loci have now been described as affecting the diabetes risk score [1]. While most of these impact insulin secretion [2], the identified variants usually lie within or between neighbouring genes and in only a few cases have the causal gene(s) been firmly established [3, 4].

Chromosome 15q hosts a risk locus close to the *VPS13C*, *C2CD4A* and *C2CD4B* genes [5], which is associated with impaired proinsulin processing. Deletion of *Vps13c* (which encodes a lipid transporter [6]) selectively from the pancreatic beta cell [7] has little effect on glucose homeostasis in the mouse. This finding argues that the other genes at this locus contribute towards the effect(s) of risk variants in humans.

Previous expression quantitative trait locus (eQTL) studies have demonstrated altered expression of *VPS13C* and *C2CD4A* [7], as well as *C2CD4B* [8], in islets from individuals carrying risk alleles. Recently, Kycia et al [9] reported that expression of *C2CD4B*, but not *C2CD4A* or *VPS13C*, was affected by risk alleles. However, the direction of effects of risk alleles differed between the reports, with expression lowered uniquely in females in the study of Mehta et al [7] but increased in the study by Kycia et al [9]. However, both of these studies involved relatively small sample numbers. These limitations emphasise the need for interventional studies involving gene inactivation in tractable model systems, such as rodents or fish, as an alternative means of understanding the roles of these genes in metabolic homeostasis.

**Fig. 1:**
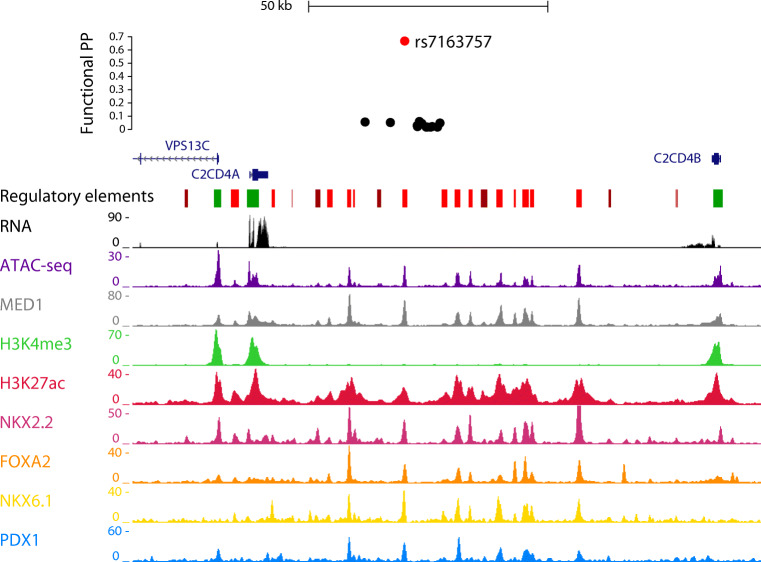
Genomic context of type 2 diabetes variants in the *VPS13C/C2CD4A/C2CD4B* locus. SNP at rs7163757 is located in an open chromatin region between *C2CD4A* and *C2CD4B*, as assessed by Assay for Transposase-Accessible Chromatin sequencing (ATAC-seq) data. Chromatin immunoprecipitation and next generation sequencing (ChIP-seq) data reveals binding sites for transcription factors involved in the development and function of beta cells, including forkhead box protein A2 (FOXA2), NK2 homeobox 2 (NKX2.2), NK6 homeobox 1 (NKX6.1) and pancreatic and duodenal homeobox 1 (PDX1). MED1, mediator complex subunit 1. Functional PP, functional posterior probability. Data from [39]

*C2CD4A* and *C2CD4B* (also called *NLF1* and *NLF2*) [10] encode low molecular mass (39 kDa) proteins of presently unknown function. Unlike the homologous *C2CD4C* gene (expressed from a distinct locus on chromosome 19 in *Homo sapiens*), neither C2 calcium dependent domain containing protein (C2CD)4A nor C2CD4B possess a canonical Ca^2+^/phospholipid-binding C2-domain [11]. A partly functional Ca^2+^-binding domain may be present in C2CD4B (see electronic supplementary material [ESM] Fig.1). Given the essential role for Ca^2+^ in the control of insulin and other hormone secretion [12], an interaction with Ca^2+^ might provide a means through which C2CD4A or C2CD4B influence these processes.

Originally described in endothelial cells as having a largely nuclear distribution, and inducible by cytokines [10], the role of C2CD4B has not been explored previously in beta cells or other metabolically relevant cell types. Nonetheless, silencing of the single homologous *C2cd4a/b* gene in the zebrafish (*Danio rerio*) led to a decrease in beta cell mass [13]. In contrast, silencing of the *Drosophila melanogaster C2CD4A* homologue, *spenito* (also known as *nito*), increased circulating levels of the insulin-like molecule IIp2HF [14]. Inactivation of *C2cd4c* has no detectable effect on pancreatic development in mice [11].

A recent study [15] has indicated that regulation of *C2cd4a* expression in islets by forkhead box O1 (FOXO1) may be important for the control of insulin secretion. Thus, animals inactivated selectively in the beta cell for *C2cd4a* displayed abnormal insulin secretion in response to exposure to glucose plus arginine (the effects on secretion stimulated by glucose alone were not reported), as well as glucose intolerance in vivo and abnormal expression of beta cell signature and ‘disallowed’ genes [16]. However, these studies used the *Ins2*-dependent rat insulin promoter (RIP) to drive *Cre* expression, a strategy complicated by off-target recombination and ectopic expression of human growth hormone [17].

Here, we use the more direct approach of inactivating *C2cd4b* and *C2cd4a* globally in the mouse, and of deleting their homologue, *C2cd4a*, in the developing zebrafish (*D. rerio)*, to explore the role of these genes in glucose homeostasis.

## Methods

For detailed Methods and the reagents list, please refer to the ESM Methods.

### Mouse generation

*C2cd4a* (C2cd4a-Del1724-EM1-B6N) and *C2cd4b* (*C2cd4b*^*em2Wtsi*^) mouse strains were generated at the International Mouse Phenotyping Consortium (IMPC; www.mousephenotype.org/data/genes/MGI:3645763 and www.mousephenotype.org/data/genes/MGI:1922947, respectively; accessed November 2020), using CRISPR/Cas9. In vitro fertilisation was performed into super-ovulated C57BL/6 females, producing mixed B6N/B6J offspring, which were subsequently inter-crossed. Lean and fat mass were measured using an EchoMRI Quantitative Whole Body Composition analyser (Zinsser Analytic, USA) on unanaesthetised animals. The high-fat and -sucrose diet (HFD) contained 58% fat and 25% carbohydrate (catalogue no. D12331; Research Diets, New Brunswick, NJ, USA), given from 6 weeks of age.

### Glucose homeostasis

Animals were fasted overnight prior to experiments. For IPGTTs, glucose (1 g/kg body weight) was injected into the abdomen. In OGTTs, glucose (2 g/kg body weight) was administered directly into the gut via oral gavage. Blood glucose levels were recorded using an automatic glucometer (Accucheck; Roche Diagnostics, Burgess Hill, UK). For insulin tolerance tests, animals were fasted for 5 h prior to experiments. Insulin was injected into the abdomen: Male and female mice (both *C2cd4b-*null and WT) on the regular chow (RC) diet were injected with a 1 or 0.75 U/kg body weight of insulin, respectively. HFD-fed male and female mice were injected with 1.5 or 0.75 U/kg body weight of insulin, respectively. Blood glucose levels were measured post injection at the time points indicated. To measure insulin secretion in vivo, animals were fasted overnight and glucose (3 g/kg body weight) was injected into the abdomen. Blood insulin levels were measured using an Ultra-Sensitive Mouse Insulin ELISA Kit (90,080; Crystal Chem, Zaandam, the Netherlands).

### Measurement of circulating hormone levels

For assessment of follicle-stimulating hormone (FSH) and luteinising hormone (LH), gonadectomy was conducted under isoflurane anaesthesia [18]. Plasma levels of testosterone and oestradiol (E2) were determined by ELISA (Enzo Life Sciences, Exeter, UK, and BioVision, Cambridge BioScience, Cambridge, UK, respectively) 2 weeks later [18].

### Insulin secretion from isolated islets

Islet isolation [19] and measurements of insulin secretion [20] were measured as described previously, using ten size-matched islets incubated in triplicate in HEPES-buffered Krebs-Ringer medium (KREBH) and either 3 mmol/l glucose, 17 mmol/l glucose or 20 mmol/l KCl at 37°C. Insulin was measured using an Insulin Ultra-Sensitive Kit (Mercodia, Uppsala, Sweden).

### Generation of C2CD4A and C2CD4B FLAG- and -green fluorescent protein-tagged constructs

Human *C2CD4A* and *C2CD4B* cDNA sequences were cloned in-frame into plasmid P3XFLAG-CMV-14 (Addgene, www.addgene.org) to provide a C-terminal 3×FLAG epitope tag. Green fluorescent protein (GFP)-tagged proteins were generated by inserting the human *C2CD4A* and *C2CD4B* cDNA sequences into the C-terminus of GFP using plasmid pEGFP-C1 (Addgene).

### C2CD4A/B intracellular translocation

INS1(832/13) cells [21] were grown on coverslips and transfected with either GFP-tagged C2CD4A, C2CD4B or synaptotagmin-1 (Syt1)-containing constructs. At 24 h post transfection, cells were incubated for 1 h at 37°C with aerated KREBH solution. Cells were confirmed to be mycoplasma free prior to experiments.

### Study approval

All mouse in vivo procedures were conducted in accordance with the UK Home Office Animal (Scientific Procedures) Act of 1986 (Project licence PA03F7F0F to IL) and approved by the Imperial College Animal Welfare and Ethical Review Body. All zebrafish work was approved by the ethical committee of the University of Liège (protocol no. 13-1557) or under European Union and German laws (Tierschutzgesetz), and with the approval of the TU Dresden and the Landesdirektion Sachsen (approval licence number: TVV 45/2018).

### Zebrafish maintenance and generation of transgenic lines

Zebrafish (*D. rerio*) were obtained from the Zebrafish International Resource Center (Zfin, Eugene, OR, USA) or from the European Zebrafish Resource Center at Karlsruhe Institute of Technology (www.ezrc.kit.edu/), and then bred at the host laboratory (University of Liège [ULg], Liège, Belgium, or Universität Dresden, Dresden, Germany, respectively). Genetically modified zebrafish were developed at the host laboratories. Zebrafish were raised and cared for according to standard protocols [22]. The transgene rs7163757C-cfos:eGFP was constructed by introducing a 1303 bp region carrying the genome-wide association study (GWAS)-identified gene variant rs7163757-C upstream of a c-fos minimal promoter driving enhanced GFP (EGFP) expression (pGW_cfos-EGFP) [23, 24] via Gateway LR recombination (Invitrogen, Thermo Fisher Scientific, Waltham, MA, USA). The transgene was injected into zebrafish embryos and GFP expression patterns were analysed during development, in adulthood and in offspring, using a confocal microscope.

### Whole-mount in situ hybridisation and immunohistochemistry on zebrafish embryos

Double fluorescent whole-mount in situ hybridisations were performed as previously described [25] with the antisense RNA probe for the different genes prepared as described [26]. Immunohistochemistry on whole-mount embryos was performed as previously described [27]. The proteins analysed were GFP, insulin, glucagon and somatostatin. Images were acquired with a confocal microscope and processed with ImageJ (https://imagej.nih.gov/ij/; accessed December 2019) and figureJ (https://imagej.net/FigureJ).

### Pericardial glucose injection and live imaging in zebrafish larvae

Glucose injection and live imaging of zebrafish primary islets were performed as described previously [28].

### Blood glucose measurements in adult zebrafish

Adult zebrafish were fasted for 24 h before glucose measurements. For post-prandial measurements, fasted fish were fed with live brine shrimp. Blood was collected from euthanised fish with a microcapillary needle and glucose levels were measured using a glucometer.

### Immunofluorescence analysis of pancreatic slices

Mouse pancreases were dissected at 24 or 25 weeks of age. Pancreases were fixed in 4% (wt/vol.) paraformaldehyde and embedded in paraffin for the measurement of insulin and glucagon via immunofluorescence.

### Homogeneous time-resolved fluorescence (HTRF) assay

The Insulin Ultra-Sensitive Kit (ref. 62IN2PEH; Cisbio, Codolet, France) was used according to the manufacturer’s instructions to measure released or total insulin levels in mouse samples. Each sample was measured in duplicate and incubated with europium cryptate and XL665 antibodies overnight before measuring the Fӧrster resonance energy transfer (FRET) efficiency.

### Intracellular free [Ca^2+^] measurements

Mouse islets were isolated and incubated with fluo2-AM (10 μmol/l; Teflabs, Austin, TX, USA) diluted in a KREBH buffer containing 3 mmol/l glucose. Imaging was performed essentially as described previously [29].

### Whole-cell voltage-clamp electrophysiology

Measurements were performed on single beta cells isolated from wild-type (WT) and *C2cd4b*-knockout mice. Starting from a holding potential of −80 mV, and voltage-dependent Ca^2+^ currents (VDCC) currents were generated through application of sequential 10 mV depolarising steps ranging from −70 mV to 70 mV (500 ms).

### Sample preparation for RNA sequencing

Islets from five male mice/genotype at 22 weeks of age on regular chow diet were used for RNA purification. Generation of double-stranded cDNA and library construction were performed using NEBNext Ultra II Directional RNA Library Prep Kit for Illumina (NEB, Hitchin, UK). Sequencing was performed by the Imperial BRC Genomics Facility (Imperial College London, UK) as 75 bp paired end reads on a HiSeq4000 according to Illumina specifications. Data are archived in the GEO repository (www.ncbi.nlm.nih.gov/geo/query/acc.cgi?acc=GSE152576).

### Quantitative reverse transcription PCR

Quantitative reverse transcription PCR (RT-qPCR**)** was performed in triplicate using SYBR Green PCR Master Mix (Applied Biosystems, Foster City, CA, USA) and primers for mouse *C2cd4a* and β-actin genes.

### Immunofluorescence and imaging for subcellular localisations

Rodent (MIN6 [15], INS1[832/13] [21]) or human (EndoCβH1 [30]) beta cell lines (previously confirmed as being mycoplasma free) were cultured for 12 h or 24 h post transfection (with C2CD4A- or C2CD4B-FLAG tagged constructs) and fixed in 4% (wt/vol.) paraformaldehyde (Sigma-Aldrich, Gillingham, Dorset, UK) before being incubated with anti-FLAG antibodies. Images were collected by a spinning disk microscope and a confocal inverted microscope.

### Immunoprecipitation and mass spectrometry

Mycoplasma-free MIN6 cells grown in standard culture conditions [31] were transfected in duplicate with FLAG-tagged-C2CD4A or -C2CD4B expressing plasmids or with a FLAG tag-only expressing plasmid using Lipofectamine 2000 (Thermo Fisher Scientific). Immunoprecipitation and affinity purification-MS (AP-MS) analysis were performed, as previously described [32], with minor modifications. The mass spectrometry proteomics data have been deposited to the ProteomeXchange repository (www.ebi.ac.uk/pride/archive/projects/PXD021597).

### Statistical analysis

Blinding was carried out for all mouse in vivo experiments. Data were analysed using GraphPad Prism 8.0 (San Diego, CA, USA; www.graphpad.com/scientific-software/prism/, accessed 1 December 2019). A *p*-value <0.05 was considered significant.

## Results

### *C2CD4A* and *C2CD4B* expression in mouse and human islets

Human C2CD4A and C2CD4B are 83% homologous. Like their murine homologues, the two human genes are predicted to have evolved from a common ancestor (see phylogenetic tree, ESM Fig. 2a). Conservation of genomic architecture (synteny) at this locus argues for direct homology between the human and murine forms of each gene. The zebrafish (*D. rerio*) possesses two *C2cd4*-like genes homologous to *H. sapiens* and *Mus musculus*: *C2cd4a* and *C2cd4c* (ESM Fig. 2a)*.* Scrutiny of gene expression databases (http://Biogps.org, accessed March 2020), and previous publications [33, 34], reveals approximately tenfold higher levels of *C2cd4b* than *C2cd4a* mRNA in mouse islets and purified mouse beta cells (ESM Table 1). In contrast, roughly equal levels of *C2CD4A* and *C2CD4B* mRNA are present in human islets [35] and purified beta cells [36]. Expression of both genes was detected in the pituitary in both human and mice (data source: GTEx data, analysed in The Human Protein Atlas; www.proteinatlas.org/ENSG00000198535-C2CD4A/tissue and www.proteinatlas.org/ENSG00000205502-C2CD4B/tissue; accessed November 2020). *C2cd4a* and *C2cd4b* were both upregulated in pancreatic islets of high-fat-diet--fed DBA2J mouse model of diabetes following 30 and 90 days of the diet vs RC-fed mice (ESM Fig. 3a,b) [37]. No evidence of *C2CD4A* or *C2CD4B* upregulation was observed in the islets of individuals with type 2 diabetes vs normoglycaemic control participants [38].

Examination of the human *VPS13C/C2CD4A/C2CD4B* locus revealed multiple regulatory elements (Fig. 1), consistent with recent findings [9]. A SNP, rs7163757, has recently been shown by fine mapping as the likely causal variant for type 2 diabetes risk at this locus [9]. Correspondingly, CRISPR activation (CRISPRa) or CRISPR interference (CRISPRi) at the rs7163757 site has the most marked effects on neighbouring genes [39], consistent with this being the effector variant (SNP). To explore in more detail the potential role of the enhancer around the diabetes risk variant rs7163757 [9] and whether it may play a role in gene expression in disease-relevant tissues (notably the islet and brain/pituitary), we introduced a reporter bearing 1303 bp nucleotides of the human sequence into the zebrafish genome, controlling the production of GFP from a minimal c*Fos* promoter (ESM Fig. 2b). Expression was restricted to the endocrine pancreas and brain at all stages (ESM Fig. 2c,e), and was detected in all islet cell types, most strongly in delta cells (ESM Fig. 2d). No significant differences were seen in the pattern of expression in embryos expressing either the C- or the T variants, with 10% (8/80 or 10/100, respectively) of embryos showing fluorescence in the pancreas.

Whole-mount fluorescent in situ hybridisation of 30 h-post-fertilisation (hpf) embryos revealed that endogenous *c2cd4a* is expressed in the forebrain (ESM Fig. 2f), ventral spinal cord (ESM Fig. 2g) and pancreas (ESM Fig. 2c,h). Double fluorescent in situ hybridisation revealed presence of the *c2cd4a* transcript in *sst2*^+^, *ins*^+^ and *gcgb*^+^ cells (ESM Fig. 2d,i–k).

### Role of *c2cd4a* in the zebrafish larvae

Zebrafish possess a single gene, *C2cd4a* (formerly *c2cd4ab*), that is homologous to the two mammalian counterparts (ESM Fig. 2a). As a convenient proxy for insulin secretion in the larvae of fish inactivated for *c2cd4a* or in WT controls (ESM Fig.4 a,b), glucose-stimulated Ca^2+^ dynamics were monitored in vivo by imaging the fluorescence of a gCaMP6 transgene. Fasting and postprandial blood glucose levels were comparable between WT and *c2cd4a* mutant animals (ESM Fig. 4c) and glucose-induced Ca^2+^ changes did not differ between genotypes (ESM Fig. 4d–h). These findings argue against a role for *c2cd4a* in beta cell function during zebrafish development.

### Effects of *C2cd4b* deletion on glucose homeostasis are more marked in female than male mice

**Fig. 2:**
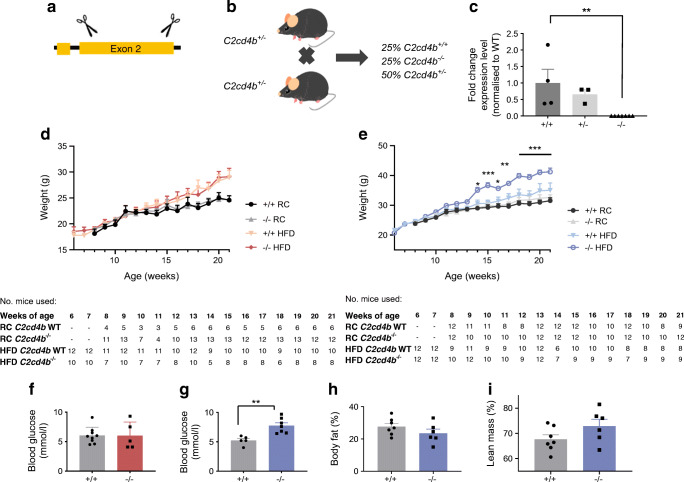
Characterisation of *C2cd4b-*null mice. (**a**) *C2cd4b* global null mice (*C2cd4b*^*em2Wtsi*^*)* were generated by the IMPC. Using CRISPR/Cas9, the encoding exon from murine *C2cd4b* (exon 2) was deleted. (**b**) *C2cd4b*^*+/−*^ (heterozygous) animals were setup as breeding pairs and the WT (*C2cd4b*^*+/+*^*)* and homozygous (*C2cd4b*^*−/−*^*)* littermates were studied. (**c**) RT-qPCR was performed on RNA from isolated islets and showed a significant decrease in *C2cd4b* mRNA levels in homozygous animals (*p*=0.0092). *C2cd4b*^*+/+*^ *n*=4, *C2cd4b*^*+/−*^ *n*=3, *C2cd4b*^*−/−*^ *n*=7. **p<0.01, unpaired Student’s *t* test. (**d**, **e**) Changes in weight of *C2cd4b*^*+/+*^ and *C2cd4b*^*−/−*^ female (**d**) and male (**e**) mice over time on an RC diet or HFD. RC: female (F)^+/+^ *n*=3–6; F^−/−^ *n*=4–13; male (M)^+/+^ *n*=8–12; M^−/−^ *n*=10–12. HFD: F^+/+^ *n*=9–12, F^−/−^ *n*=5–10–12; M^+/+^ *n*=6–12, M^−/−^ *n*=7–12. (**f**, **g**) Fasting blood glucose level in female (**f**) and male (**g**) mice on an HFD were measured at 23 weeks of age. F^+/+^ *n*=9; F^−/−^ *n*=5; M^+/+^ *n*=5; M^−/−^ *n*=7. (**h**, **i**) Percentage of body fat (**h**) and lean mass (**i**) in males maintained on an HFD at 20 weeks of age. *p<0.05, **p<0.01, ***p<0.001, mixed-effect analysis, RC-fed WT vs RC-fed mutant mice or HFD-fed WT vs HFD-fed mutant mice at each time point (**d**, **e**), or Student’s *t* test (**c**, **f**–**i**). Data were assessed for significance using an unpaired Student’s *t* test or two-way ANOVA where two genotypes were compared. Values represent means ± SEM

Given the substantially higher expression of *C2cd4b* than *C2cd4a* in mouse islets (ESM Table 1), we studied mice in which the former gene was deleted (Fig. 2a). Inter-crossing of heterozygous animals produced pups at the expected Mendelian ratio (Fig. 2b) and resulted in complete elimination of *C2cd4b* mRNA from isolated islets of *C2cd4b*^−/−^ mice (Fig. 2c). Female *C2cd4b*-null mice gained weight at the same rate as control littermates whether maintained on RC or a HFD (Fig. 2d) and no differences were apparent in fed or fasting blood glucose levels either on an RC diet (ESM Fig. 5a,c) or an HFD (Fig. 2f and ESM Fig. 5e).

Beta cell mass, as assessed by histochemical analysis of pancreatic slices, was unaffected by deletion of *C2cd4b* in both females (ESM Fig. 6a–d) and males (ESM Fig. 6e–h).

**Fig. 3:**
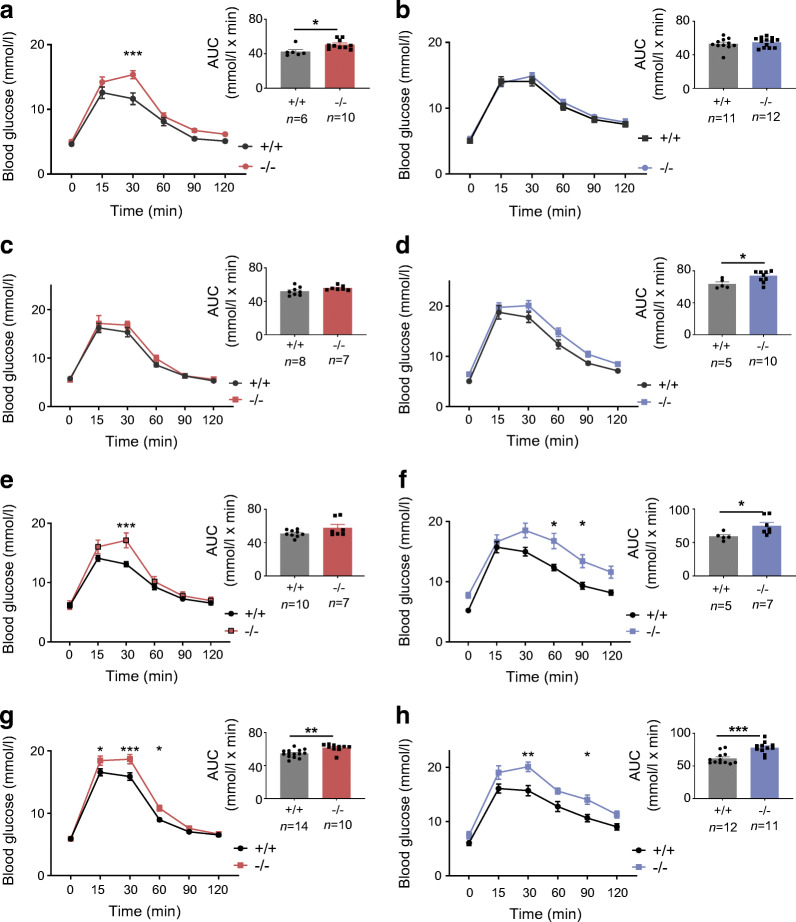
*C2cd4b*-null mice display glucose intolerance in glucose tolerance tests (IPGTTs/OGTTs). (**a**, **b**) IPGTTs were performed on female (**a**) and male (**b**) mice maintained on an RC diet, at 22 weeks of age. (**c**, **d**) OGTTs were performed on *C2cd4b*-null and WT female (**c**) and male (**d**) mice on an RC, at 20 weeks of age. (**e**, **f**) IPGTTs were performed on *C2cd4b*-null and WT female (**e**) and male (**f**) mice maintained on an HFD, at 23 weeks of age. (**g**, **h**) OGTTs were performed on *C2cd4b* female (**g**) and male (**h**) mice on an HFD, at 20 weeks of age. AUC analyses are also shown. The *n* values under bar graphs represent the number of animals used (the same number of samples were used for blood glucose and AUC graphs). *p<0.05, **p<0.01, ***p<0.001 vs mutant animal at same time point or as indicated. Blood glucose curves assessed by two-way ANOVA with Bonferroni’s multiple comparison test; AUC analyses assessed using an unpaired Student’s *t* test. Values represent means ± SEM

**Fig. 4:**
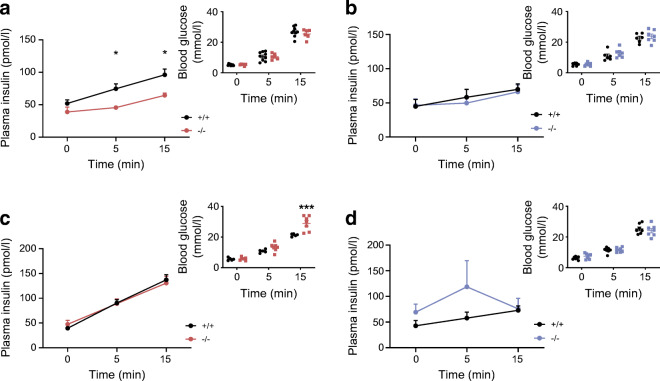
Effect of *C2cd4b* deletion on in vivo glucose-stimulated insulin secretion. (**a**–**d**) In vivo insulin levels during IPGTT in *C2cd4b*-null and WT females (**a**, **c**) and males (**b**, **d**) on an RC diet, at 23 weeks of age (**a**, **b**) or an HFD at 19 weeks of age (**c**, **d**). RC: female (F)^+/+^ *n*=9; F^−/−^ *n*=6; male (M)^+/+^ *n*=6; M^−/−^ *n*=7. HFD: F^+/+^ *n*=5; F^−/−^ *n*=7; M^+/+^ *n*= 7; M^−/−^ *n*=7. *p<0.05, ***p<0.001, WT vs mutant animal at same time point, two-way ANOVA with Bonferroni’s multiple comparison test. Values represent mean ± SEM

Intraperitoneal glucose tolerance was examined for animals maintained on an RC diet or HFD from 8 to 22 weeks of age. Females displayed abnormalities at 12, 20 and 22 weeks of age when fed an RC diet (ESM Fig. 7a,c,e,g), and at 8 and 23 weeks on an HFD (ESM Fig. 8a,c,e and Fig. 3a; *p* < 0.001 at 22 weeks on an HFD) vs control littermates. While oral glucose tolerance was normal on in RC-fed female mice vs controls (Fig. 3c), defects were observed in HFD-fed mice (Fig. 3g). For female mice on the RC diet, these changes were associated with defective insulin secretion in vivo (Fig. 4a; *p* = 0.02), as also indicated by unchanged insulin levels after glucose injection despite elevated plasma glucose levels in female knockout mice maintained on an HFD (Fig. 4c). Furthermore, insulin sensitivity was not different between *C2cd4b* null and WT female mice (ESM Fig. 9a,c).

When maintained on an RC diet, male *C2cd4b* null mice gained weight at the same rate as WT littermates. When maintained on an HFD, in contrast to females, male mutant mice gained substantially more weight from 14 weeks of age vs WT littermates (Fig. 2d,e; *p* < 0.001 betwee18 and 21 weeks of age), and had raised fasting blood glucose levels (Fig. 2g; *p* = 0.03). However, significant differences in body fat and lean mass were not apparent (Fig. 2h,i) vs WT mice. Intraperitoneal glucose tolerance was normal in males maintained on an RC diet at most ages examined, with genotype-dependent differences only at 16 weeks (ESM Fig. 7b,d,f,h and Fig. 3b). Although unaltered in younger male mice after maintenance on an HFD (ESM Fig. 8b,d,f), glucose tolerance was impaired at 23 weeks of age by *C2cd4b* deletion (Fig. 3f,h) as compared with controls.

As observed in females, insulin sensitivity (ESM Fig. 9b,d) was unaltered in male *C2cd4b*-null mice vs littermate controls.

### Effects of *C2cd4b* deletion on beta cell function in vitro

Glucose-stimulated insulin secretion was not different between islets from WT or *C2cd4b*-null female mice maintained on an RC diet (ESM Fig. 10a) but was slightly elevated in those from female null mice maintained on an HFD (ESM Fig. 10c). Likewise, in the isolated islets from female *C2cd4b*-null mice, we observed no alterations in glucose or KCl-stimulated stimulated Ca^2+^ dynamics (ESM Fig. 11a,b) or in beta cell–beta cell coupling (ESM Fig. 11c–f). Correspondingly, no changes in voltage-activated Ca^2+^ currents were apparent in patch-clamp recordings (ESM Fig. 12a–c).

In line with the above findings, massive parallel sequencing (RNA-Seq) of islets from *C2cd4b*^−^null or control animals confirmed the lowering of *C2cd4b* expression in islets of null mice vs WT mice, and revealed a significant (~75%) increase in *C2cd4a* expression (ESM Table 2), though this was not confirmed by independent qRT-PCR analysis (data not shown). However, no other mRNAs were significantly affected by *C2cd4b* deletion (ESM Table 2).

Glucose or KCl-stimulated insulin secretion from isolated islets were also unaltered in male *C2cd4b*-null mice vs littermate controls (ESM Fig. 10 b,d).

### Effects of *C2cd4b* deletion on pituitary function

**Fig. 5:**
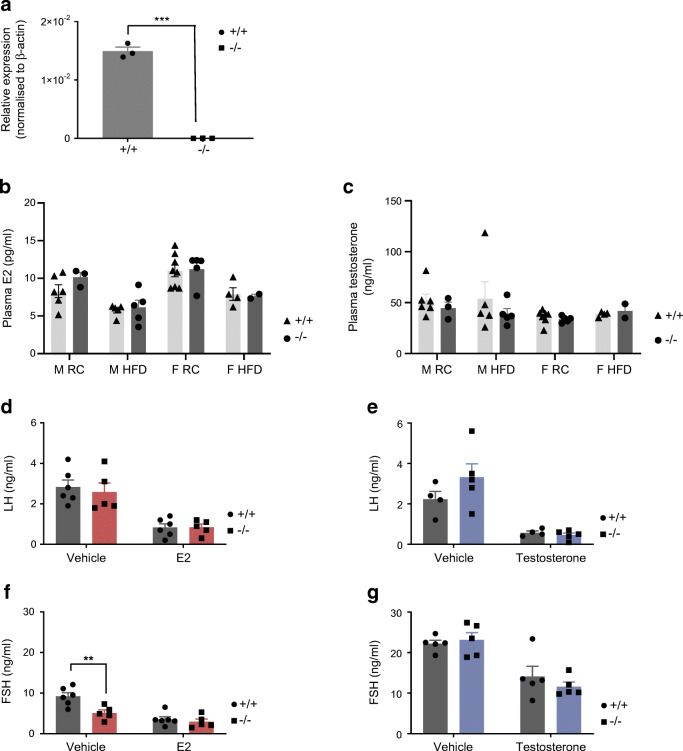
Effect of deletion of *C2cd4b* on sex hormones and hormone release from the pituitary gland. (**a**) RT-qPCR on samples from the pituitary glands shows basal level of mRNA expression of *C2cd4b* in animals deleted for this gene compared with WT mice. (**b**, **c**) Measurement of E2 and testosterone levels upon deletion of *C2cd4b* in female and male mice maintained on either an RC diet or an HFD. (**d**, **e**) Circulating LH levels in *C2cd4b*-null and WT female (**d**) and male (**e**) mice after injections with saline (154 mmol/l NaCl; vehicle), E2 or testosterone (animals were maintained on an RC diet). (**f**, **g**) Circulating FSH levels in *C2cd4b*-null and WT female (**f**) and male (**g**) mice after injections with saline (vehicle), E2 or testosterone (animals were maintained on an RC diet). Female (F)^+/+^ *n*=6, F^−/−^ *n*=5; male (M)^+/+^ *n*=4; M^−/−^ *n*=5. **p<0.01, ***p<0.001, unpaired Student’s *t* test. Values represent mean ± SEM

Given the absence of clear defects in insulin secretion in isolated *C2cd4b*-null islets, and the expression of both *C2cd4a* and *C2cd4b* in the pituitary (data source: GTEx data, analysed in The Human Protein Atlas; www.proteinatlas.org/ENSG00000198535-C2CD4A/tissue and www.proteinatlas.org/ENSG00000205502-C2CD4B/tissue; Fig. 5a), we next assessed whether deletion of this gene might affect the production of sex hormones and, thus, provoke gender-specific differences in glucose homeostasis. E2 and testosterone levels were unaltered in both male and female *C2cd4b*-null mice maintained on an RC diet or HFD vs WT animals (Fig. 5b,c). In order to remove the negative feedback loop through which hormones secreted from the gonads repress FSH and LH release from the pituitary gland, animals between 12 and 15 weeks of age were gonadectomised prior to these experiments. Compared with WT littermates, female *C2cd4b*-null mice displayed ~50% lower circulating FSH levels, with no difference in LH levels (Fig. 5d,f; *p* = 0.003). No differences in LH or FSH levels were apparent between WT and *C2cd4b*-null male mice (Fig. 5e,g).

### *C2cd4a*-null mice display no metabolic abnormalities

To determine whether *C2cd4a* inactivation might also have an impact on glucose homeostasis, we next examined metabolic phenotypes in male and female *C2cd4a*-null mice (ESM Fig. 13a–c). In contrast to their *C2cd4b*-null counterparts, *C2cd4a*-null mice displayed no evident metabolic abnormalities up to 22 weeks of age, with neither weight gain, glucose homeostasis nor insulin secretion differing between WT and null mice for either sex (ESM Fig. 13d–i). Likewise, as measured in vitro, glucose and KCl (depolarisation)-stimulated insulin secretion were unaltered in islets isolated from male *C2cd4a*-null mice (ESM Fig. 13j).

### C2CD4A but not C2CD4B C2 domains support Ca^2+^-dependent intracellular translocation

We next sought to explore the mechanism(s) through which C2CD4B or C2CD4A may influence beta cell (and, potentially, pituitary gonadotroph) function. Both proteins have been suggested to lack a functional C2 domain [11], consistent with a reported localisation in the nucleus in COS7 cells [10]. In contrast to earlier findings reporting nuclear subcellular localisation, when overexpressed as GFP- or FLAG- tagged chimaeras in rodent (MIN6, INS1[832/13]) or human (EndoCβH1) beta cell lines, C2CD4A and C2CD4B were found at the plasma membrane and in the cytosol and the nucleus (ESM Fig.14 and ESM Fig.15). In the majority of cells, C2CD4A and C2CD4B were primarily localised to the cytoplasm and nucleus. Co-localisation with readily identified intracellular sub-compartments, including the secretory granule (insulin), trans-Golgi network (TGN46), endosome/lysosome (LAMP1) or endoplasmic reticulum (ER; KDEL), was not apparent in the above cell lines (ESM Fig. 16; data only shown for MIN6 cells but results were consistent in all three cell lines).

**Fig. 6:**
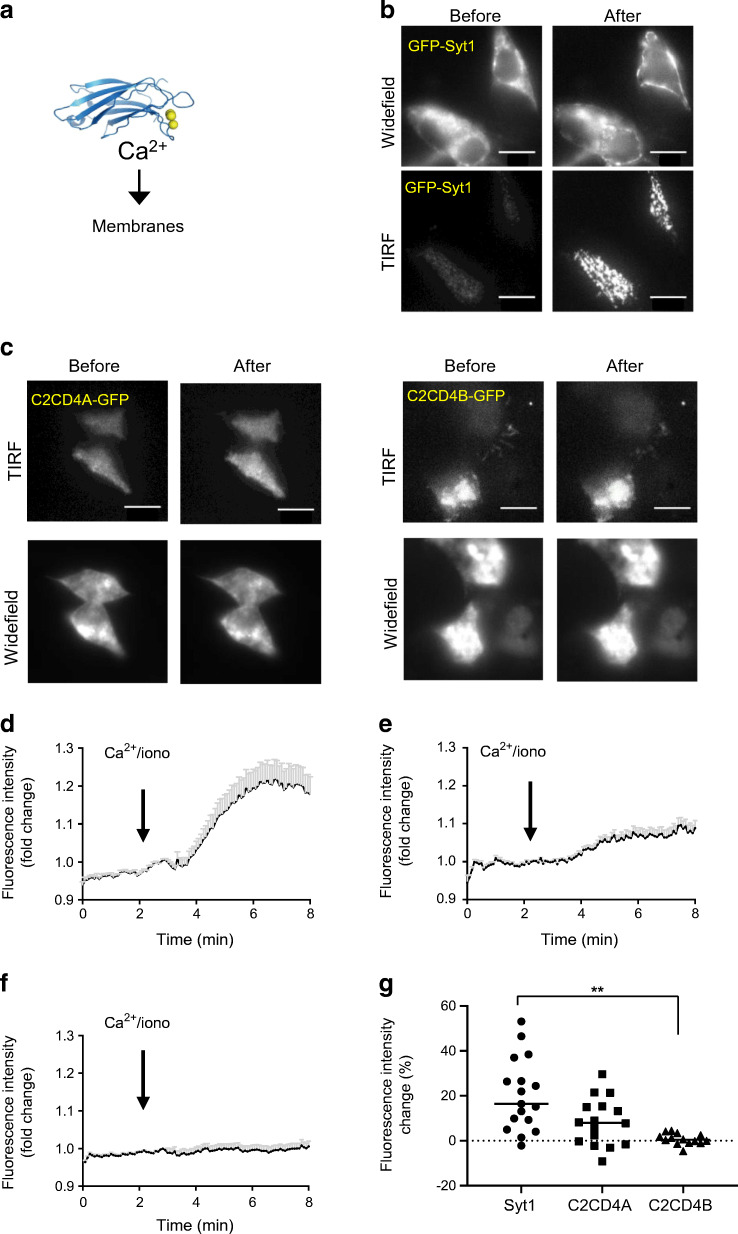
Changes in C2CD4A and C2CD4B localisation in response to increased intracellular [Ca^2+^]. (**a**) Schematic showing the principle of Ca^2+^-dependent translocation of C2 domains to the plasma membrane. The ribbon diagram reproduced from [57] under the terms of the Creative Commons Attribution 4.0 International License (http://creativecommons.org/licenses/by/4.0/), which permits unrestricted use, distribution, and reproduction in any medium. (**b**) Localisation of GFP-Syt1 in cells incubated in KREBH buffer with 3 mmol/l glucose, shown before and after an increase in intracellular Ca^2+^ levels (achieved by addition of 50 ng/ml ionomycin), obtained by simultaneous wide-field and TIRF image acquisition (positive control). (**c**) Localisation of GFP-tagged C2CD4A and -C2CD4B before and after addition of ionomycin, obtained by simultaneous wide-field and TIRF image acquisition. (**d**, **f**) Time courses for the translocation of the GFP-tagged proteins, Syt1 (**d**), C2CD4A (**e**) or C2CD4B (**f**) obtained by TIRF imaging before and after addition of ionomycin (iono). (**d**–**f**) Solid black lines represent mean; SEM shown by grey bars. (**g**) Assessment of fluorescence intensity change (%) reveals an increase in C2CD4A intensity at the plasma membrane after the imposed increase in intracellular Ca^2+^ levels, which was similar to that observed for Syt1 protein translocation, whilst C2CD4B translocation was significantly lower than Syt1. For each condition, *n*=3 independent experiments were performed; *n=*44 cells were tracked in Syt1, *n*=29 cells were tracked in the case of C2CD4A, and *n*=30 cells were tracked in the case of C2CD4B. Scale bars = 10 μm. **p<0.01, one-way ANOVA

The above findings suggested that the C2 domain of either protein may bind to Ca^2+^ and contribute to localisation at, and/or shuttling between, subcellular compartments in living cells. To test this hypothesis, we explored phospholipid-dependent recruitment of these proteins to the plasma membrane in INS1(832/13) beta cells [21] expressing either a control construct, in which Syt1 (bearing five C2 domains) was fused to GFP [40] or equivalent C2CD4A or C2CD4B constructs (N-terminal linkage). In response to an increase in intracellular free Ca^2+^, provoked by 50 μmol/l extracellular Ca^2+^ and the calcium ionophore ionomycin (50 ng/ml), Syt1–GFP translocated from intracellular (likely ER-bound) sites to the plasma membrane. This movement was readily visualised by simultaneous live-cell wide-field and total internal reflection of fluorescence (TIRF) imaging (Fig. 6a,b,d). A similar, but smaller change in the localisation of C2CD4A–GFP in response to Ca^2+^ was also observed. In contrast, no response was detected for C2CD4B-GFP (Fig. 6c,e–g).

### Identification of C2CD4A and C2CD4B binding partners by mass spectrometry

The above experiments demonstrated that C2CD4A, and possibly C2CD4B, may participate in Ca^2+^-dependent signal transduction. To gain further insight into possible mechanisms of action, we performed an unbiased proteomic screen using immunoprecipitation and mass spectrometry to identify potential binding partners. Normalising to the negative control, and ranking in order of protein abundance, we generated lists of possible interacting proteins for human C2CD4A, C2CD4B (ESM Tables 3,4) or both (ESM Table 5). MIN6 cells transfected with human C2CD4A or C2CD4B were used for this analysis, given the low transfection efficiency of human-derived EndoCβH1 cells. Interacting partners included proteins involved in Ca^2+^ binding (torsin-2A [TOR2A] and EF-hand calcium-binding domain-containing protein 5 [EFCAB5; [41] and www.genecards.org/), NF-κB signalling (sequestosome-1 [SQSTM1] and programmed cell death protein 11 [PDCD11]) and protein trafficking (proprotein convertase subtilisin/kexin type 9 [PCSK9], neural precursor cell expressed, developmentally down-regulated 4, E3 ubiquitin protein ligase [NEDD4], Ras-proximate-1 [Rap1] and GTPase-activating protein [GAP2]). Receptor-type tyrosine-protein phosphatase-like N (PTPRN; insulinoma-associated protein 2 [IA-2]) and PTPRN2; phogrin/IA-2β/islet cell antigen 512 [ICA512]) bound to both C2CD4A and C2CD4B. These protein tyrosine phosphatase-like transmembrane proteins are granule-resident and implicated in granule trafficking and exocytosis [42].

## Discussion

The overall aim of this study was to examine the biological roles of *C2cd4b* and *C2cd4a* in vivo, focusing on the pancreatic beta cell and pituitary gland. These questions have been pursued using mouse and fish knockout models and relevant cell lines. In contrast to earlier findings [15], we observed that global *C2cd4a* deletion in the mouse exerted no effects on insulin secretion in vitro or in vivo. These findings are consistent with the considerably (tenfold) lower expression of *C2cd4a* than *C2cd4b* in mouse islets and mouse-derived beta cell lines (ESM Table 1), though we should emphasise that no attempt was made here to quantify protein (rather than mRNA) levels. The reasons for the differences between the present study and that of Kuo et al [15] with respect to *C2cd4a* are presently unclear.

### Deletion of *C2cd4b* leads to weight gain in males, but defective insulin secretion in female mice

Our findings reveal a strikingly sexually dimorphic phenotype of *C2cd4b*-null mice, which is also strongly dependent upon diet. In contrast to females, male *C2cd4b* null mice displayed no evident metabolic abnormalities at most ages when maintained on an RC diet. Although mutant male mice exhibited a substantial increase in body weight on the HFD, fasting glucose and glucose tolerance were impaired only in older (>20 weeks) animals. In stark contrast, female mutant mice displayed abnormal glucose tolerance from as early as 12 weeks when fed an RC diet and 8 weeks when fed an HFD (with a tendency towards abnormal glucose tolerance [*p* > 0.05] being observed at 8 weeks in RC-fed mice), despite unaltered body weight. Non-significant changes in lean mass were observed in male *C2cd4b*-null mice on HFD, which are reminiscent of findings from the IMPC (www.mousephenotype.org/data/genes/MGI:1922947, accessed November 2020) when this line was fed an RC diet. The mechanisms underlying these changes remain unclear but are unlikely to involve elevated testosterone levels in knockout animals, since these were not observed in our study.

Although earlier human studies were not stratified by sex, we note that a statistically significant (*p* = 0.004) impact of the human type 2 diabetes-associated variant rs7172432 (which is in perfect linkage disequilibrium with rs7163757: *R*^2^ = 1.0) [15] at the *VPS13C/C2CD4A/C2CD4B* locus on waist circumference has previously been reported [43], and may be related to the alterations in body weight we observed here in male *C2cd4b*-knockout mice.

What factors underlie these sex-dependent differences in insulin secretion between WT and *C2cd4b*-null mice? Our findings suggest that beta cell-extrinsic mechanisms, possibly involving circulating factors, contribute to (and may even be the drivers of) altered insulin secretion in the living animal. Potential contributors are changes in FSH levels [44, 45] in female *C2cd4b*-null mice, reflecting altered expression of the gene in the pituitary. Decreased FSH production is expected, in turn, to decrease circulating oestrogen levels. We note, however, that measurements of oestrogen are complicated in fertile mice due to fluctuations in the oestrous cycle [46]. Positive actions of oestrogen on beta cell insulin content (via oestrogen receptor α) and secretion (via oestrogen receptor β) are well known [44, 47] and may thus underlie the weaker insulin secretion in *C2cd4b*-knockout mice. Consistent with a requirement for sexual maturity, no differences were apparent in beta cell function between genotypes in the living fish embryo (ESM Fig.4) at stages where differences in circulating sex hormones are not anticipated, and fasting blood glucose levels did not differ between WT and knockout fish at adult stages. These findings are in contrast to earlier studies in zebrafish larvae using oligonucleotide-mediated *C2cd4a* gene knockdown [13], which reported alterations in beta cell mass.

Such sexual dimorphism on the impact of variants at this locus, however, has not been reported in human GWAS data [48, 49]. One possible explanation is that dimorphism in mice reflects well-known differences in the response of males and females to HFD [50]. More likely, in our view, is that in rodent islets (and pituitary; see below), *C2cd4b* expression predominates over *C2cd4a*, while in humans, *C2CD4A* and *C2CD4B* expression are comparable. Consequently, in humans, changes in both *C2CD4A* and *C2CD4B* may mediate the effect of the GWAS signal for type 2 diabetes, dampening a sexually dimorphic effect of changes in *C2CD4B* expression.

Might altered genetic risk for type 2 diabetes in humans result from changes in *C2CD4A* or *C2CD4B* expression in the brain? Importantly, high levels of expression of C2CD4A and C2CD4B in the pituitary (GTEx data, The Human Protein Atlas; www.proteinatlas.org/ENSG00000198535-C2CD4A/tissue and www.proteinatlas.org/ENSG00000205502-C2CD4B/tissue; accessed November 2020) are consistent with this view. Correspondingly, an eQTL for *C2CD4A* is reported for rs7163757 in the pituitary (www.gtexportal.org/home/snp/rs7163757#sqtl-block, accessed November 2020), with a (non-significant) tendency towards decreased expression of *C2CD4A* and, more strongly, *VPS13C* in carriers of C (risk allele) vs T alleles.

### Intracellular signalling by *C2cd4a* and *C2cd4b*

Our observation that neither C2CD4A nor C2CD4B are localised exclusively in the nucleus, as previously reported in COS7 cells [10], islets and MIN6 cells [15], was unexpected, but implies a more dynamic role for both C2CD4A, and possibly C2CD4B, in intracellular signalling. We note that the present study explored the subcellular distribution of the *H. sapiens* protein, rather than the *M. musculus* homologue examined previously [15], providing a potential explanation for these differences. Interestingly, predictions from the primary structure [11] indicate that neither C2CD4A nor C2CD4B (human or mouse) possesses a C2 domain with a bona fide Ca^2+^-binding site [51, 52]. Nevertheless, we provide a direct demonstration of Ca^2+^-dependent recruitment of C2CD4A to the plasma membrane (Fig. 6), whereas C2CD4B would appear to exert its function independently of Ca^2+^ binding.

What signalling mechanism(s) may lie downstream of C2CD4A (or C2CD4B)? The interacting protein PTPRN2 (also known as phogrin and IA-2β) is of particular interest [42]. Inactivation of PTPRN2/phogrin, a secretory granule-localised protein [53], leads to defective insulin secretion in mice [54, 55]. Importantly, double knockout of PTPRN2/phogrin and the closely-related *Ptprn* (*IA-2*) gene leads to defective FSH and LH production and female infertility [54], implying an important role in the anterior pituitary. An interaction between C2CD4A or C2CD4B and PTPRN2 in the pituitary (and possibly the beta cell) may, therefore, contribute to the effects of altered expression on diabetes risk.

### Limitations of the study

The present work was undertaken using animals on a mixed C57BL6N:C57BL6J background and future studies are required to confirm the findings here in pure-bred strains [56]. The impact of *C2cd4b* deletion on fertility, and on the role of *C2cd4a* and *C2cd4b* in the pituitary, remain to be explored, as does the contribution of changes in food intake or energy expenditure to differences in body weight. More work will also be necessary to determine whether *c2cd4a*-null zebrafish display beta cell deficiency.

Restrictions imposed by the coronavirus disease-2019 (COVID-19) pandemic prevented further explorations of these points during the present study.

## Supplementary information

ESM 1 (PDF 1711 kb)

## Data Availability

The datasets generated and/or analysed during the current study are available in the Biorxiv repository, (www.biorxiv.org/content/10.1101/2020.05.18.099200v1). RNA-Seq (GSE152576) and proteomics (PXD021597) data have been deposited to GEO (www.ncbi.nlm.nih.gov/geo/query/acc.cgi?acc=GSE152576) and ProteomeXchange (www.ebi.ac.uk/pride/archive/projects/PXD021597) repositories, respectively.

## Abbreviations

C2CD: C2 calcium dependent domain containing protein
E2: Oestradiol
eQTL: Expression quantitative trait locus
ER: Endoplasmic reticulum
FSH: Follicle-stimulating hormone
GFP: Green fluorescent protein
GWAS: Genome-wide association study
HFD: High-fat and -sucrose diet
IA-2: Insulinoma-associated protein 2
IMPC: International Mouse Phenotyping Consortium
KREBH: HEPES-buffered Krebs-Ringer medium
LH: Luteinising hormone
PTPRN: Receptor-type tyrosine-protein phosphatase-like N
RC: Regular chow
Syt1: Synaptotagmin-1
TIRF: Total internal reflection of fluorescence
WT: Wild-type
