# Supplementary material for: Sexually dimorphic roles for the type 2 diabetes-associated C2cd4b gene in murine glucose homeostasis
Source: Diabetologia. 2021 Jan 25;64(4):850–64. doi: 10.1007/s00125-020-05350-x (PMC7829492; doi:10.1007/s00125-020-05350-x)
Supplement: Supplementary file 1 — (PDF 1711 kb) [file 125_2020_5350_MOESM1_ESM.pdf]

Electronic supplementary material (ESM): Mousavy-Gharavy *et al*

ESM Research Design and Methods

Reagents

Mouse monoclonal anti-FLAG antibody (1:1000 vol/vol, Sigma-Aldrich, F1804-200UG), guinea-pig polyclonal anti-insulin (1:1000 vol/vol., DAKO, Agilent Technologies, Stockport, U.K.; ready to use, IR002), mouse monoclonal anti-glucagon antibody (1:1000 vol/vol, Sigma-Aldrich, G2654-100UL), rabbit polyclonal antibody anti-TGN46 (1:500 vol/vol, Abcam, Cambridge, U.K. Ab16059), rat monoclonal anti-LAMP-1 antibody (1:200 vol/vol, Santa Cruz, Dallas, TX, U.S.A., Sc-19992) were used for immunostaining of fixed cells and/or paraffin sections. Alexa Fluor 488 goat anti-guinea-pig (A-11073), Alexa Fluor 532 goat anti-rabbit (A11009) and Alexa Fluor 488 goat anti-mouse (A11029) were used as secondary antibodies from Invitrogen (Thermo Fisher Scientific, Waltham, MA, U.S.A.) all 1:1000 vol/vol. All the washes were carried out in PBS-Triton (0.1% vol/vol) and the antibodies were diluted and incubated in PBS-0.1%(vol/vol)Triton-1%(wt/vol)BSA.

Mouse generation

C2cd4a (C2cd4a-Del1724-EM1-B6N) and *C2cd4b* (*C2cd4b*^{em2Wtsi}) mouse strains were generated at the International Mouse Phenotyping Consortium (IMPC), using CRISPR/Cas9. *In vitro* fertilisation was performed into super-ovulated C57BL/6 females, producing mixed B6N/B6J offspring, which were subsequently inter-crossed.

C2cd4a and *C2cd4b* null strains were maintained on a C57BL/6N background. In the *C2cd4a* strain, 1742 bp were deleted from exon 2 and in *C2cd4b* strain, exon 2 was deleted.

Heterozygous animals were used as breeding pairs. Genotyping was carried out according to the IMPC protocols.

Mice were housed in a pathogen-free facility with a 12-hour light/dark cycle and had free access to standard mouse chow diet and water. Animals fed with high-fat and -sucrose diet containing 58% fat and 25% carbohydrate, provided from Research Diets, (New Brunswick, N.J. U.S.A.) Cat. No. D12331. Animals were maintained on and had free access to this diet from 6 weeks of age. Lean and fat mass were measured using an EchoMRI Quantitative Whole Body Composition analyser (Zinsser Analytic, USA) on unanaesthetised animals.

Glucose homeostasis

Animals were fasted overnight prior to experiments. For intraperitoneal glucose tolerance tests (IPGTT), glucose (1 g/kg body weight) was injected into the abdomen. In oral glucose tolerance test (OGTT), glucose (2 g/kg body weight) was administered directly into the gut via oral gavage. Blood glucose levels were recorded using an automatic glucometer (Accucheck, Roche Diagnostics, Roche Diagnostics. Burgess Hill, U.K.). For insulin tolerance tests, animals were fasted for 5 h prior to experiments. Insulin was injected into the abdomen (concentrations indicated in the main text). Blood glucose levels were measured post-injection at the time points indicated. To measure insulin secretion *in vivo*, animals were fasted overnight and glucose (3 g/Kg body weight) injected into the abdomen. Blood insulin levels were measured using an Ultra-Sensitive Mouse Insulin ELISA Kit (Crystal Chem, Zaandam, Netherlands, 90080).

Measurement of circulating hormone levels

For assessment of follicle-stimulating hormone (FSH) and luteinizing hormone (LH), gonadectomy was conducted under isoflurane anaesthesia, and aseptic conditions, according to standard protocols. Animals received post-operative analgesia, and were allowed to recover for two weeks before collection of blood for hormone analysis (1). Plasma levels of testosterone and oestradiol (E2) were determined by ELISA (Enzo Life Sciences, Exeter, U.K. and BioVision, Cambridge BioScience, Cambridge, U.K.).

Insulin secretion from isolated islets

Islets were isolated after pancreatic distension with collagenase, essentially as previously described (2). Insulin secretion was measured as described (3) with 10 size-matched islets in triplicate incubated in Krebs-HEPES-bicarbonate (KREBH) solution (3) containing either: 3 mM glucose, 17 mM glucose or 20 mM KCl at 37°C with gentle shaking. Insulin was measured using an Insulin Ultra-Sensitive Kit (Mercodia, Upsala, Sweden).

Generation of C2CD4A and C2CD4B -FLAG and -GFP tagged constructs

Human *C2CD4A* and *C2CD4B* cDNA sequences were cloned in-frame into plasmid P3XFLAG-CMV-14 (Addgene, www/addgene.org) to provide a C-terminal 3xFLAG epitope tag. Green fluorescent protein (GFP)-tagged proteins were generated by inserting the human *C2CD4A* and *-B* cDNA sequences into the C-terminus of GFP using plasmid pEGFP-C1 (Addgene).

C2CD4A/B intracellular translocation

Cells were grown on coverslips and transfected with either GFP-tagged C2CD4A, C2CD4B or Syt1-containing constructs. 24 h post-transfection, cells were incubated for 1 h at 37°C with aerated KREBH solution.

Study approval

All mouse *in vivo* procedures were conducted in accordance with the UK Home Office Animal (Scientific Procedures) Act of 1986 (Project license PA03F7F0F to I.L.) and approved by Imperial College Animal Welfare and Ethical Review Body. All zebrafish work was approved by the ethical committee of the University of Liège (protocol #13-1557) or under European Union and German laws (Tierschutzgesetz), and with the approval of the TU Dresden and the Landesdirektion Sachsen (approval license number: TVV 45/2018).

Zebrafish maintenance and generation of transgenic lines

Zebrafish (*Danio rerio*) were raised and cared for according to standard protocols (4). All animal work has been conducted according to national guidelines and all animal experiments described herein were approved by the ethical committee of the University of Liège (protocol number 13-1557).

The transgene rs7163757C-cfos:eGFP was constructed by introducing a 1303 bp region carrying the GWAS rs7163757-C upstream of a c-fos minimal promoter driving EGFP (pGW_cfos-EGFP) (5,6) via Gateway® LR recombination (Invitrogen, Life technologies. Purified rs7163757C-cfos:eGFP transgene was injected into 1–2-cell zebrafish embryos and the resulting GFP expression pattern analysed at different time points during development (F0 analysis). The fluorescent injected fish were raised to adulthood and the offspring screened for fluorescence. GFP expression pattern of the transgenic line obtained was tested by whole

mount immunohistochemistry as described in (7) using chicken anti-GFP (Aves Labs, Davis, CA, U.S.A., 1:1000), guinea pig anti-Insulin (Dako,1:500) and fluorescently conjugated AlexaFluor antibodies (Invitrogen). Fluorescent images were acquired with a Leica SP5 confocal microscope (Leica Biosystems, Milton Keynes, U.K.).

Whole mount *in situ* hybridisation and immunohistochemistry on zebrafish embryos

Double Fluorescent whole-mount *in situ* hybridizations were performed as previously described (8) with the antisense RNA probe for the different genes prepared as described (9). Immunohistochemistry (IHC) on whole mount embryos has been performed as described (7). The antibodies used were, chicken anti-GFP (Aves lab) 1:1000, guinea pig anti-Insulin (Dako) 1:500, mouse anti-Glucagon (Sigma) 1:300, polyclonal rabbit anti-Somatostatin (MP Biomedicals) 1:300, Alexa Fluor secondary antibodies (Invitrogen). DAPI was used as nuclear staining.

Images were acquired with a Leica SP5 confocal microscope and processed with ImageJ (https://imagej.nih.gov/ij/; downlad Dec. 2019) and FigureJ (https://imagej.net/FigureJ).

Pericardial glucose injection and live imaging in zebrafish larvae

To record glucose-stimulated calcium dynamics in β cells *in vivo*, heterozygous animals carrying a 1kb deletion in the *C2cd4a* promoter were in-crossed. The progeny was sorted for the presence of the *Tg(ins:GCaMP6s,cryaa:RFP)*^{tud202Tg} reporter, which was previously introduced in the background of the *C2cd4a* promoter deletion. Glucose injections and live imaging were performed blindly for the genotype of the larvae. After imaging, each larva was genotyped, and the genotype was assigned to the corresponding images.

Glucose injection and live imaging of zebrafish primary islets were performed as described previously (10). The embryos were treated with 0.003% (200μM) 1-phenyl-2-thiourea to inhibit pigmentation, starting from 1 dpf. At 4.5 dpf, the larvae were anaesthetized with 0.4 g/l tricaine (MS-222) and mounted in 1% low melting point agarose containing 0.4 g/l tricaine in 35 mm glass bottom petri dishes (MatTek Corporation, Ashland, MA, U.S.A.). After the agarose solidified, embryo water containing 0.4 g/l tricaine was added onto the embryos.

Live imaging was performed on a Zeiss LSM 780 (Carl Zeiss Ltd, Cambourne, Cambs, U.K.) inverted confocal microscope with a 40x water lens (Zeiss C-Apochromat 40x/1.2 W autocorr M27). The GCaMP6s signal was acquired using the 488 laser line. Image acquisition was performed using a single focal plane, with a speed of 150 ms per frame (6.66Hz) for 400 frames (60 seconds), and an XY resolution of 512 x 512 pixels.

Blood glucose measurements in adult zebrafish

Adult zebrafish were fasted for 24 h before glucose measurements (Males and females). For post prandial measurements, fasted fish were fed with live brine shrimp. After 1 hour, the fish were euthanized and blood was collected with a microcapillary needle. Each needle was washed with heparin beforehand to avoid blood coagulation. Accu-Chek Aviva blood glucose meter (Roche Diagnostics, Roche Diagnostics. Burgess Hill, U.K.) was used to measure blood glucose.

Immunofluorescence on pancreatic slices

Animals were dissected at 24 or 25 weeks of age. Pancreata were fixed in 4% [wt/vol.] paraformaldehyde (PFA, Sigma-Aldrich) diluted in phosphate-buffered saline (PBS). Samples

sent to the histology facility at Imperial College London were embedded in paraffin and sectioned at 5 µm thickness. Each section was 150 µm apart from the previous section. Primary antibodies (guinea-pig anti-insulin ready to use (Dako): not diluted, mouse anti-glucagon (Sigma-Aldrich): 1:1000), diluted in PBS (containing: 0.25% BSA, 0.25% Triton X-100), were applied overnight at 4°C. Slides were incubated with secondary antibodies (Alexa Fluor 488 goat anti-guinea-pig, Invitrogen, 1:1000, Alexa Fluor 532 goat anti-rabbit, Invitrogen,1:1000, DAPI, Roche Diagnostics, 1:5000) for 2 h at room temperature (RT). ProLong Dimond Antifade Mountant, Life Technologies was used for mounting. An inverted widefield microscope with LED illumination Zeiss Axio Observer microscope (Zeiss Axio Observer Z1), from the Imperial College FILM facility, was used to collect images.

Homogeneous Time Resolved Fluorescence (HTRF) Assay

Insulin Ultra-Sensitive Kit (Cisbio, Codolet, France, ref. 62IN2PEH) was used according to the manufacturer's instructions to measure released or total insulin levels. In order to assess the final dilutions for samples, a test with several dilutions was carried out before measuring all samples collected. Each sample was measured in duplicate and incubated with europium cryptate and XL665 antibodies overnight before measuring the Förster Resonance Energy Transfer (FRET) efficiency.

Intracellular free [Ca^{2+}] measurements

Imaging was performed essentially as described (11). In brief, 24 h after islet isolation, 20 islets/acquisition were incubated for 45 min. in fluo2-AM (10 µM; Teflabs, Austin, TX, U.S.A.) diluted in a KREBH buffer solution containing 3 mM glucose. A Nipkow spinning disk head microscope was used to capture the fluorescent signals. Islets were maintained at 35°C to 36°C and continuously irrigated with KREBH buffer solution aerated with 95% O_2

and 5% CO_2. On average, 8-10 islets were imaged in each field of view. Two acquisitions were performed per animal. Images were analysed using ImageJ software (URL: https://imagej.nih.gov/ij/index.html) by measuring the fluorescence over time. The Pearson product moment correlation analysis was performed for every possible cell pair to assess inter-cellular connectivity (10).

Whole-cell voltage-clamp electrophysiology

Wild type control and *C2cd4b* knockout mouse islets were dispersed into single cells by gently titration for 1 minute in 0.005% trypsin and cultured overnight in RPMI-1640 medium (RPMI) with 11 mM glucose supplemented with 15% fetal bovine serum (FBS), 100 IU·ml^{-1} penicillin, and 100 mg·ml^{-1} streptomycin at 37°C, 5% CO_2. Patch electrodes (3-4 MΩ) were backfilled with intracellular solution containing (mM) 102.0 CsCl, 10.0 TEA-Cl, 10.0 EGTA, 3.0 Na_2ATP, and 5.0 HEPES (pH 7.25 adjusted by CsOH). Voltage-clamp electrophysiology was performed on β-cells in extracellular buffer containing (mM) 119.0 NaCl, 4.7 KCl, 2.0 $CaCl_2$, 1.2 $MgSO_4$, 1.2 KH_2PO_4, 10.0 HEPES, and 17.0 glucose (pH 7.35 adjusted by NaOH). After forming a tight seal between the patch pipette and β-cell (seal resistance > 1 GΩ), whole-cell access was established and the bath solution was exchanged (3 minutes; 2 mL/minute flowrate) with extracellular buffer containing (mM) 82.0 NaCl, 5.0 CsCl, 30.0 $CaCl_2$, 1.0 $MgCl_2$, 0.1 EGTA, 20.0 TEA-Cl, 0.1 tolbutamide, and 17.0 glucose (pH 7.35 with NaOH). Starting from a holding potential of -80, VDCC currents were generated through application of sequential 10 mV depolarizing steps ranging from -70 to 70 mV (500 ms); membrane potential was held at -80 mV for 7.5 seconds between each voltage step. Linear leak currents were subtracted using a P/4 protocol. VDCC currents were normalized to cell capacitance and normalized peak VDCC currents plotted as a function of applied voltage.

Sample preparation for RNA sequencing

Isolated islets from 5 male mice/genotype at 22 weeks of age on RC were used for RNA purification. DNase treatment was performed using TURBO DNase (Invitrogen) according to the manufacturer's instructions. RNA quantity and integrity were assessed using an RNA 6000 Nano Kit (Agilent) and an Agilent 2100 Bioanalyzer. mRNA enrichment was achieved from 0.8-1 µg of total RNA using a NEBNext Poly(A) mRNA Magnetic Isolation Kit (NEB, Hitchin, U.K.). Generation of double stranded cDNA and library construction were performed using NEBNext Ultra II Directional RNA Library Prep Kit for Illumina (NEB). NeBNext Multiplex Adapters (NEB) was used to perform ligation of the adapters. Each library was subsequently size selected with SPRIselect Beads (Beckman Coulter). The Adaptor ligated DNA was PCR amplified using NEBNext Ultra II Q5 Master Mix and Universal i5 and i7 primers provided in the NEBNext Kits.

Sequencing was performed by the Imperial BRC Genomics Facility as 75bp paired end reads on a HiSeq4000 according to Illumina specifications. FASTQ files were generated for each sample (5 WT and 4 null mice) and initial data quality checks of the raw sequence data were performed. Reads were then mapped to the mouse transcriptome (GRCm38, cDNA and ncRNA) using Salmon (12). Total RNA profiles were consistent and generated ~20-40 million reads mapping to Ensembl genes per sample. DESeq2 (v1.20.0) (13) with DESeq2-default normalization method and adjusted p-value threshold <0.1 was used for differential expression analysis in R (www.r-project.org; accessed 1/12/2019) using relevant BioConductor packages (14). Data have been archived to GEO (accession number GSE152576).

RT-q-PCR

Total RNA was extracted from islets (70-150) using TRIzol according to the manufacturer's instructions (Invitrogen) and treated with 3U/ul DNAse I (Thermo Fisher). RNA (100-400 ng) was reverse transcribed using the High-Capacity cDNA reverse transcription kit (Applied Biosystems, Foster City, CA, U.S.A.) including random primers. Real-time PCR was performed in triplicate using a SYBR Green PCR master mix (Applied Biosystems) and primers for *C2cd4a* and beta actin genes.

C2cd4a forward primer GCCATGAGATGCACAGAGACT; reverse primer GGCCAAGAAGCTGCAAGTG; beta actin forward primer CACTGTCGAGTCGCGTCC; reverse primer TCATCCATGGCGAACTGGTG.

Immunofluorescence and imaging for sub-cellular localisations

Cells were cultured on coverslips and 12 or 24 h post transfection were fixed in 4% [wt/vol.] PFA (Sigma-Aldrich), and incubated with primary antibody (mouse anti-FLAG, Sigma-Aldrich, 1:1000). Samples were incubated with secondary antibody (Alexa Fluor 488 anti-mouse, Invitrogen, 1:1000) for 2 h at room temperature. A Nikon ECLIPSE Ti spinning disk microscope was used to collect images. A x60 oil immersion objective was used for localisation and co-localisation experiments. For co-localisation with ER experiments a confocal inverted Zeiss LSM-780 microscope was used to capture images.

Immunoprecipitation and mass spectrometry

MIN6 cells grown in standard culture conditions (15) were transfected in duplicates with 1 µg FLAG-tagged C2CD4A, C2CD4B or with FLAG tag only expressing plasmids using Lipofectamine 2000 (Thermo Fisher). Immunoprecipitation and AP-MS analysis was performed as previously described (16) with minor modifications.

Samples were processed with on-bead digestion using 1M urea followed by Trypsin Gold (Promega, Madison, WI, U.S.A.), acidified with TFA and de-salted using reversed-phase spin tips (Glygen Corp, Columbia, MD, U.S.A.). Dried peptides were subjected to LC-MS/MS analysis using an Ultimate 3000 nano HPLC coupled to a Q-Exactive mass spectrometer (Thermo Scientific) via an EASY-Spray source. Peptides were loaded onto a trap column (Acclaim PepMap 100 C18, 100µm × 2cm, Thermo Fisher Scientific) at 8µL/min in 2% acetonitrile, 0.1% TFA. Peptides were then eluted on-line to an analytical column (Acclaim Pepmap RSLC C18, 75µm × 75cm). Data was processed using the MaxQuant (17) software platform (v1.6.2.3), with database searches carried out by the in-built Andromeda search engine against the Uniprot *Mus musculus* database (downloaded – 4th January 2018, entries: 83,123). A reverse decoy database approach was used at a 1% FDR for peptide spectrum matches and protein identifications. Search parameters included: maximum missed cleavages set to 2, variable modifications of methionine oxidation, protein N-terminal acetylation, asparagine deamidation and cyclisation of glutamine to pyroglutamate. Label-free quantification was enabled with an LFQ minimum ratio count of 2.

Proteins were filtered for those containing at least 2 razor and unique peptides, identified in all replicates with a minimum of a 2-fold increase in abundance compared with control (FLAG-tag only) immunoprecipitates and ranked by average intensity across replicates.

The mass spectrometry proteomics data have been deposited to the ProteomeXchange Consortium via the PRIDE (18) partner repository with the dataset identifier PXD021597.

Statistical analysis

Data were analysed using GraphPad prism 8.0. (San Diego, CA, U.S.A., https://www.graphpad.com/scientific-software/prism/; updated Dec 1st, 2019). p-values <0.05 were considered significant.

ESM Table 1

<table>
<tr><th>Gene</th><th>Expression</th><th>Tissue</th><th>Publication</th><th>Expression</th><th>Tissue</th><th>Publication</th></tr>
<tr><td>Murine C2cd4a</td><td>6.98 (RPKM)</td><td rowspan="2">β</td><td rowspan="2">Benner et al. (19)</td><td>5.5 (RPKM)</td><td rowspan="2">Mouse pancreatic islets</td><td rowspan="2">Kone et al. (20)</td></tr>
<tr><td>Murine C2cd4b</td><td>71.6 (RPKM)</td><td>40.7 (RPKM)</td></tr>
<tr><td>Human C2CD4A</td><td>57.9 (RPKM)</td><td rowspan="2">β</td><td rowspan="2">Benner et al. (19)</td><td>18.5 (TPM)</td><td rowspan="2">β</td><td rowspan="2">Blodgett et al. (21)</td></tr>
<tr><td>Human C2CD4B</td><td>52.8 (RPKM)</td><td>15.7 (TPM)</td></tr>
</table>

ESM Table 1. Expression of *C2CD4A* and *C2CD4B* in human and mouse islets and β-cells. While both *C2CD4A* and *C2CD4B* are expressed at the same level in human β-cells, in mouse *C2cd4b* is more predominantly expressed compared to *C2cd4a*. β=FACS purified β-cells.

ESM Table 2

Gene name	Base Mean	log2FoldChange	Fold Change	p value	Adjusted p value
C2cd4b	1139.51706	-2.415152232	0.187485089	1.36E-44	3.81E-40
C2cd4a	118.027192	0.814689614	1.758919692	2.86E-06	0.040127223
Cpsf1	1904.77052	-0.578923095	0.669463314	7.06E-06	0.066105298
C3	152.141699	0.741957418	1.672443436	3.45E-05	0.241943108
Rpph1	140.612162	-0.703996659	0.613869264	5.77E-05	0.323789867
Gm45322	417.49085	0.620112657	1.536995197	0.00011	0.504901364
mt-Nd4l	3597.92578	0.680785322	1.603012107	0.000126	0.504901364
Emid1	454.688167	-0.607583874	0.656294899	0.000149	0.524402308
Crb1	108.03916	-0.647844838	0.638233023	0.000268	0.766730644
Gm10076	52.4812133	-0.547066149	0.684410525	0.000273	0.766730644

ESM Table 2. Effect of *C2cd4b* deletion on islet gene expression. The gene expression levels from isolated islets of four male mice per genotype were assessed by RNA-seq. Table presents gene expressions from high to low adjusted p values. The top ten genes with the highest adjusted p-values were selected in the above table. A significant reduction in *C2cd4b* expression levels was observed comparing islets from *C2cd4b* null to *C2cd4b* WT mice. *C2cd4a* expression was increased in *C2cd4b* null mice.

ESM Table 3

Name	Unique peptides	Average intensity	FC over control	Function
C2cd4b;C2cd4a	4	1692900000	infinity	
60S ribosomal protein L36 (Rpl36)	7	408547500	2.31651774	Ribosomal protein
Bone morphogenetic protein 1 (Bmp1)	14	323217500	4.528805647	Formation of cartilage in vivo
Clusterin beta/alpha chain (Clu)	6	242922500	infinity	Basic biological events such as cell death, tumour progression, and neurodegenerative disorders
Sequestosome-1 (Sqstm1)	4	238032500	4.041972241	Scaffolding/adaptor protein in concert with TNF receptor-associated factor 6 to mediate activation of NF-kB
Glucagon;Glicentin (Gcg)	2	149391750	infinity	Glucagon, glucose homeostasis
Ttc13	9	142825000	2.147951497	A protein coding gene, unknown function
Procollagen-lysine,2-oxoglutarate 5-dioxygenase 3 (Plod3)	8	137387500	infinity	Catalyses the hydroxylation of lysyl residues in collagen-like peptides
Hyaluronan and proteoglycan link protein 4 (Hapln4)	4	129820000	infinity	Extracellular matrix structural constituent and hyaluronic acid binding
Torsin-2A;Prosalusin;Salusin-beta (Tor2a)	3	107971000	infinity	Increases intracellular [Ca^{2+}], induces cell mitogenesis
U5 small nuclear ribonucleoprotein (Snrnp40)	4	103415000	infinity	Component of the U5 small nuclear ribonucleoprotein (snRNP) particle
MAP7 domain-containing protein 1 (Map7d1)	3	102242500	infinity	Structural molecule activity
Nucleolar protein 14 (Nop14)	4	91338750	infinity	Pre-18s rRNA processing and small ribosomal subunit assembly
DNA topoisomerase 2-beta (Top2b)	4	88047000	infinity	Chromosome condensation, chromatid separation, and the relief of torsional stress
Proprotein convertase subtilisin/kexin type 9 (Pcsk9)	6	86949000	infinity	Processes protein and peptide precursors trafficking
Receptor-type tyrosine-protein phosphatase N2 (Ptprn2)	4	82972000	infinity	Required for normal accumulation of secretory vesicles in hippocampus, pituitary and pancreatic islets

ESM Table 3. C2CD4A interacting proteins detected by mass-spectrometry. Top 15 predicted interacting proteins for C2CD4A in MIN6 cells. The given function(s) of the identified protein was adopted from GeneCards.org.

ESM Table 4

Name	Unique peptides	Average intensity	FC over control	Function
Neuroendocrine convertase 2 (Pcsk2)	18	2188050000	2.365537387	Is a protease that processes protein and peptide precursors trafficking through the secretory pathway
Renin receptor (Atp6ap2)	8	1197847500	4.624034467	Associated with the transmembrane sector of the V-type ATPases
Carboxypeptidase E (Cpe)	12	852947500	2.488765327	Involved in the biosynthesis of peptide hormones and neurotransmitters, including insulin
C2cd4a; C2cd4b	4	852000000	infinity	
Clusterin beta chain;Clusterin alpha chain (Clu)	6	364380000	infinity	Basic biological events such as cell death, tumour progression, and neurodegenerative disorders
N-acetylglucosamine-1-phosphotransferase subunit gamma (Tce7;Gnptg)	8	361852500	3.757036085	Sub-unit of an enzyme that catalyses the formation of mannose 6-phosphate (M6P) in the Golgi apparatus
Importin-5 (Ipo5)	5	274114500	infinity	Nucleoplasmic transport of proteins containing NLS
Carbohydrate sulfotransferase 11 (Chst11)	4	238460000	2.402356026	Catalyses the transfer of sulphate to position 4 of the N-acetylgalactosamine (GalNAc) residue of chondroitin
Glucagon (Gcg)	2	217025000	infinity	Glucagon, glucose homeostasis
Sequestosome-1 (Sqstm1)	4	211023500	3.868217344	Regulates activation of the nuclear factor kappa-B (NF-kB) signalling pathway
Bone morphogenetic protein 1 (Bmp1)	14	200115000	3.837129646	Formation of cartilage in vivo
Procollagen-lysine,2-oxoglutarate 5-dioxygenase 3 (Plod3)	8	195605000	infinity	Autophagy receptor required for selective macro-autophagy (aggrephagy).
Hyaluronan and proteoglycan link protein 4 (Hapln4)	4	144687500	infinity	A protein involved in Integrin Pathway and ERK Signalling
Receptor-type tyrosine-protein phosphatase N2 (Ptprn2)	4	107998500	infinity	Required for normal accumulation of secretory vesicles in hippocampus, pituitary and pancreatic islets (22–24)

ESM Table 4. C2CD4B interacting proteins detected by mass-spectrometry. Top 15 predicted interacting proteins for C2CD4B in MIN6 cells. The given function(s) of the identified protein was adopted from GeneCards.org.

ESM Table 5

Name	Unique peptides	FC over count	Function
**C2 calcium dependent domain containing 4**	4	inf	
**Clusterin; (Clu)**	6	inf	Basic biological events such as cell death, tumour progression, and neurodegenerative disorders
**Procollagen-lysine,2-oxoglutarate 5-dioxygenase 3 (Plod3)**	8	inf	Autophagy receptor required for selective macroautophagy (aggrephagy).
**Glucagon (Gcg)**	2	inf	Glucagon, glucose homeostasis
**Hyaluronan and proteoglycan link protein 4 (Hapln4)**	4	inf	A protein involved in Integrin Pathway and ERK Signalling
**Receptor-type tyrosine-protein phosphatase N2;Protein-tyrosine-phosphatase (Ptprn2)**	4	inf	Required for normal accumulation of secretory vesicles in hippocampus, pituitary and pancreatic islets (22–24)
**Importin-5 (Ipo5)**	5	inf	Nucleoplasmic transport of proteins containing NLS
**EF-hand calcium-binding domain-containing protein 5 (Efcab5)**	2	inf	Calcium-binding protein
**E3 ubiquitin-protein ligase UBR5 (Ubr5)**	8	inf	E3 ubiquitin-protein ligases, targeting specific proteins for ubiquitin-mediated proteolysis
**Heat shock 70 kDa protein 13 (Hspa13)**	5	inf	A member of the heat shock protein 70 family and is found associated with microsomes
**Proprotein convertase subtilisin/kexin type 9 (Pcsk9)**	6	inf	Regulation of cell proliferation or differentiation.
**Alpha-internexin (Ina)**	2	inf	Neurofilaments comprise the exoskeleton and they functionally maintain the neuronal calibre
**Receptor-type tyrosine-protein phosphatase-like N (Ptprn)**	6	inf	Normal accumulation of secretory vesicles in hippocampus, pituitary and pancreatic islets (22–24)

ESM Table 5. C2CD4A and C2CD4B interacting proteins detected by mass-spectrometry. Top 12 predicted interacting proteins with both C2CD4A and C2CD4B in MIN6 cells. The given function of the identified protein was adopted from GeneCards.org.

ESM Fig. 1

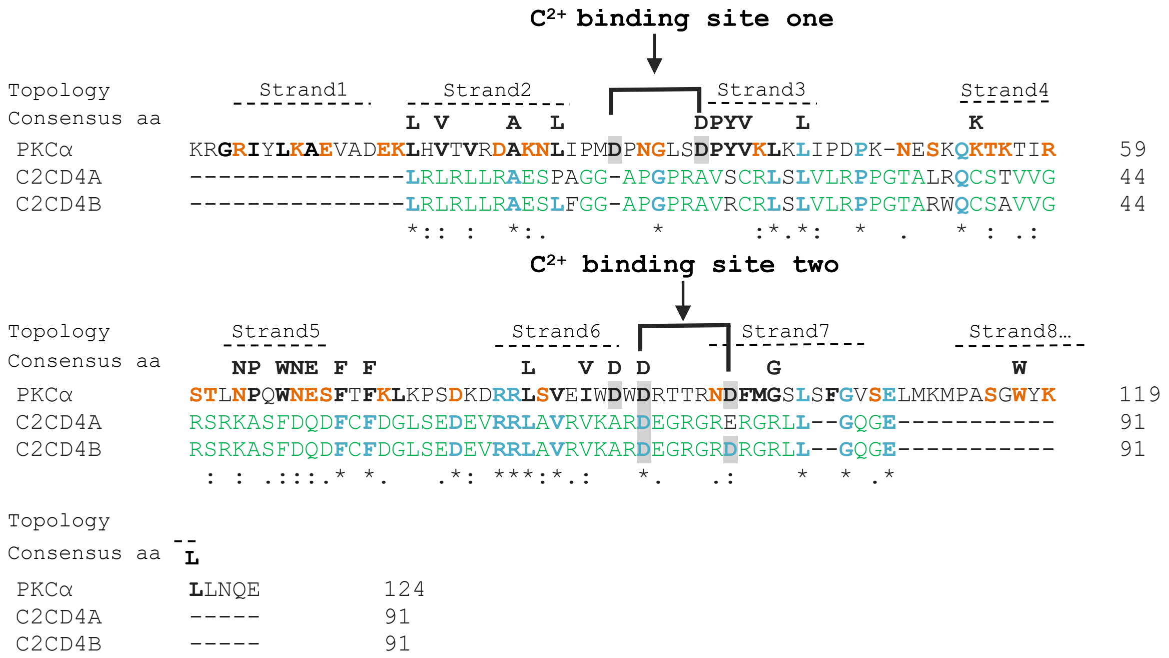

ESM Figure 1. Amino acid sequence alignment of the C2 domains of hPKCα, hC2CD4A and hC2CD4B. The secondary structure is schematically indicated above the sequence as hashed lines, which indicate β-strands in synaptotagmin, a Ca^{2+} sensor protein. The consensus sequence present in >50% of the C2 domains from 65 previously published C2 domains are indicated in bold on top (PKC structure and topology adopted from Nalefski *et al*, 1996). Amino acids shown in bold and black are non-polar or aromatic while if shown in orange they are polar or charged amino acids in consensus C2 domains. Highlighted in grey are side chains which are predicted to coordinate Ca^{2+} in synaptotagmin and PKCα. The amino acids shown in blue are conserved in PKCα; shown in green are identical aa sequences between C2CD4A and C2CD4B. (Figure generated using sequence alignment on NCBI).

ESM Fig. 2

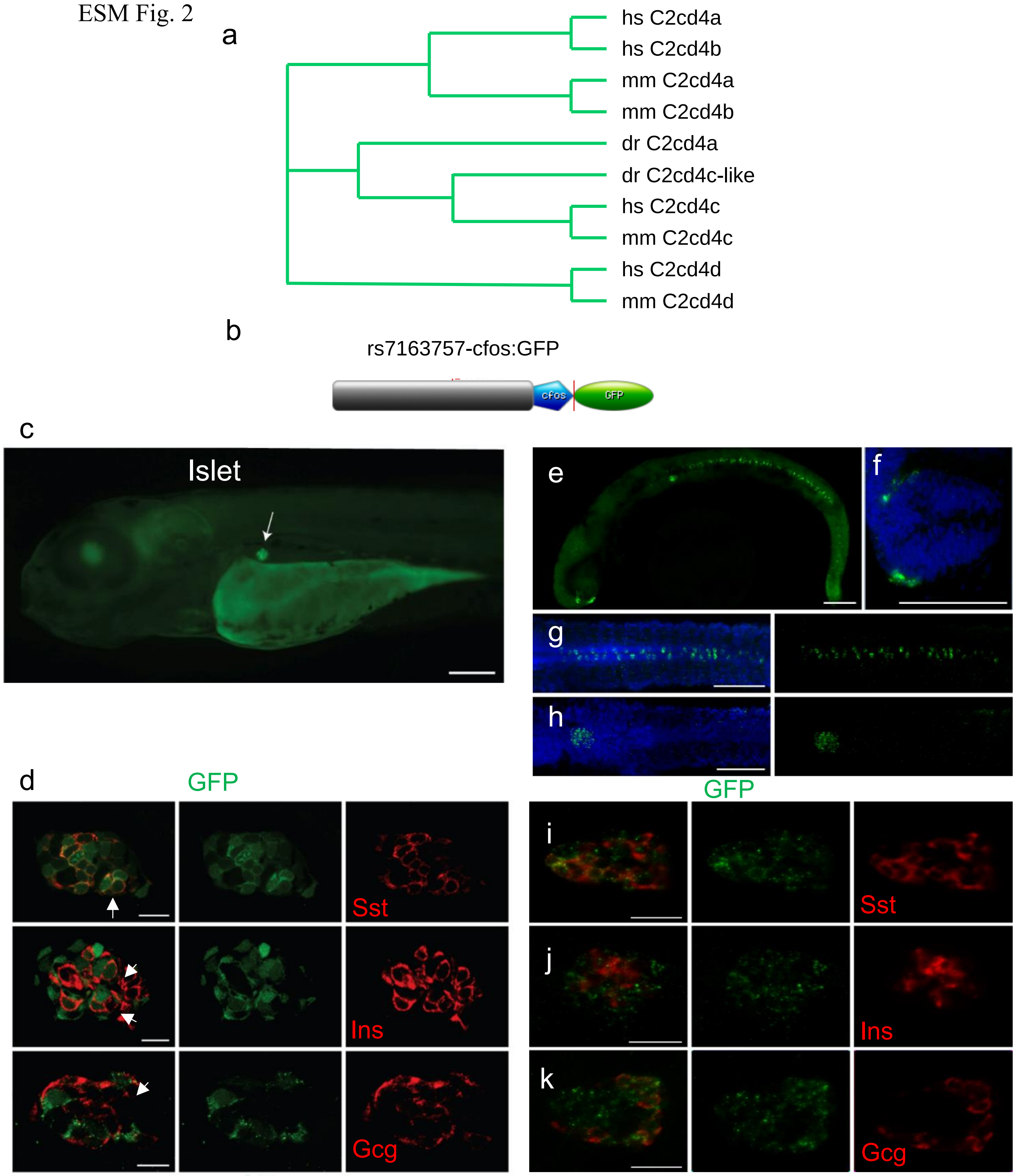

ESM Figure 2. The C2CD4 family and control of expression in zebrafish. a. The Phylogenetic tree was reproduced based on AlignX program of Vector NTI, using the full-length amino acid sequences of vertebrate members of the C2CD4 family. The numbers in parenthesis are their calculated evolutionary distances. dr-C2cd4a (ENSDARG00000061416, chr 25, previously called dr-C2cd4ab), dr-C2cd4c-like (ENSDARG00000079876.3, CU855878, chr 22). The rs7163757 region is able to direct the expression of GFP specifically in the endocrine pancreas and neurons in zebrafish. b. Schematic representation of the reporter construct rs7163757-cfos:GFP where GFP is under the control of a minimal promoter c-fos and of 1303 bp of the enhancer region containing the rs7163757 SNP. c. Data are shown are for the C variant. Similar findings were made with the T form. Binocular image of 4 dpf F2 transgenic line, rs7163757-cfos:GFP, revealing the expression of the GFP in the pancreas. Scale Bar : 200 µm. d. Whole-mount immunohistochemistry of 4 dpf rs7163757-cfos:GFP fish showing that GFP is expressed in the vast majority of somatostatin (sst)-positive cells, in most insulin-positive cells and in a few glucagon (gcg)-positive cells. Arrows indicate colocalization of dr-C2CD4A with the red channel. Scale Bar : 10 µm. Hs: Homo Sapiens, mm: mus musculus, dr: danio rerio. e-k: Whole-mount fluorescent *in situ* of 30 hpf embryos showing the expression profile of *dr-c2cd4a*. e-h: General view and close-up showing that *c2cd4a* is expressed in the ventral part of the forebrain (f), in scattered cells of the ventral part of the spinal cord (g) and in the pancreas (h). Scale Bar :100 µm. i-k: Double fluorescent *in situ* showing that *c2cd4a* transcript is expressed in *sst2*$^+$ cells (i), *ins*$^+$ cells (j) and *gcgb*$^+$ cells (k). Scale Bar : 20 µm.

ESM Fig. 3

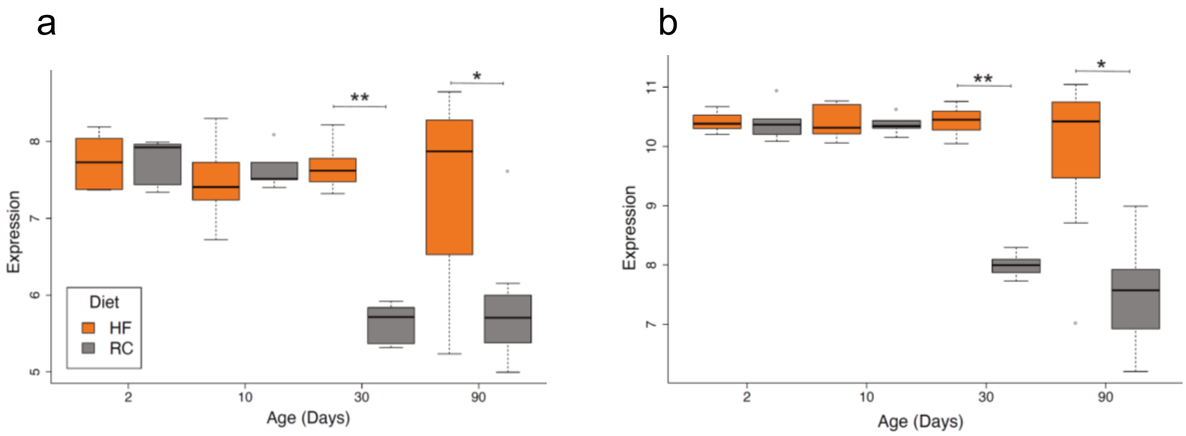

ESM Figure 3. Boxplots of *C2cd4a* and *C2cd4b* normalised expression level in pancreatic islets, see (25) for details, in DBA/2J mice fed a high fat (HF) or regular chow (RC) diet for 2, 10, 30 and 90 days. a. *C2cd4a* expression is significantly elevated in HF vs RC at 30 days (limma moderated t-test p-value=1.68e-10; Benjamini Hochberg adjusted p-value = 8.48e-9) and at 90 days (limma moderated t-test p-value=0.009; Benjamini Hochberg adjusted p-value = 0.029). b. *C2cd4b* expression is significantly elevated in HF vs RC at 30 days (limma moderated t-test p-value=4.32e-15; Benjamini Hochberg adjusted p-value = 4.58e-12) and at 90 days (limma moderated t-test p-value=0.0003; Benjamini Hochberg adjusted p-value= 0.0196). **adjusted p-value ≤0.01, *adjusted p-value ≤0.05.

ESM Fig. 4

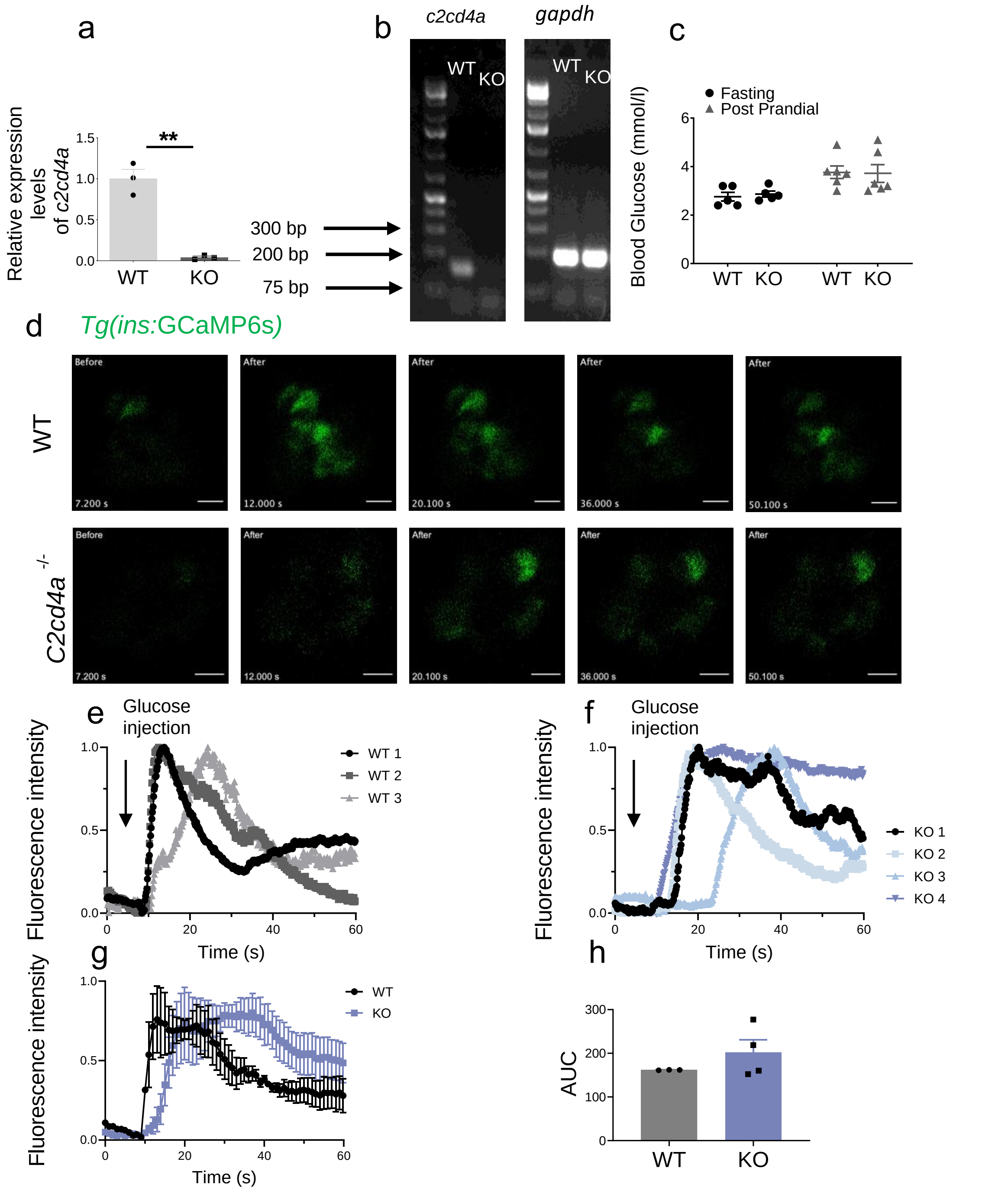

ESM Figure 4. Glucose-stimulated calcium influx is not altered by *c2cd4a* deficiency in zebrafish larvae. a. RT-q-PCR analysis of the *c2cd4a* expression in 5dpf *c2cd4a* mutant (KO) zebrafish larvae compared to wild type (WT). Relative expression was calculated with the ΔΔCt method using *gapdh* as the reference gene. Wild type expression was set at 1. b. Agarose gel electrophoresis of PCR products obtained from amplification of reverse transcribed cDNA from 5dpf *c2cd4a* mutant (KO) and wild type (WT) zebrafish larvae. Expected product sizes were 139 bp for *c2cd4a* and 179 bp for gapdh. c. Assessment of blood sugar levels in adult fish showed comparable fasting and postprandial levels between WT and KO animals, indicating that beta cell function was unperturbed. d. Representative time series showing single confocal planes of islets in WT and *C2cd4a* null zebrafish larvae (upper and lower panel respectively) before and after pericardial injection of 25 mM glucose. The larvae express the genetically encoded Ca^{2+} indicator GCaMP6s (green) in their beta cells, which exhibits an increase in fluorescence upon glucose stimulation in both WT and *C2cd4a* KO larvae. Glucose injection took place after 7 sec of imaging. e-g. Plots showing normalized islet GCaMP6s fluorescence intensity: $(F_t - F_{Min})/(F_{max} - F_{min})$, over time for different islets from WT (WT, n=3), *C2cd4a* null larvae (KO, n=4) and the averages of both (g) with area under the curve $([(F_t - F_{Min})/(F_{max} - F_{min})]$ x min), quantified in h. The arrows indicate the time of glucose injection, showing a consistent increase in GCaMP6s fluorescence upon glucose injection in WT and *C2cd4a* null larvae. Scale bars= 10 µm.

ESM Fig. 5

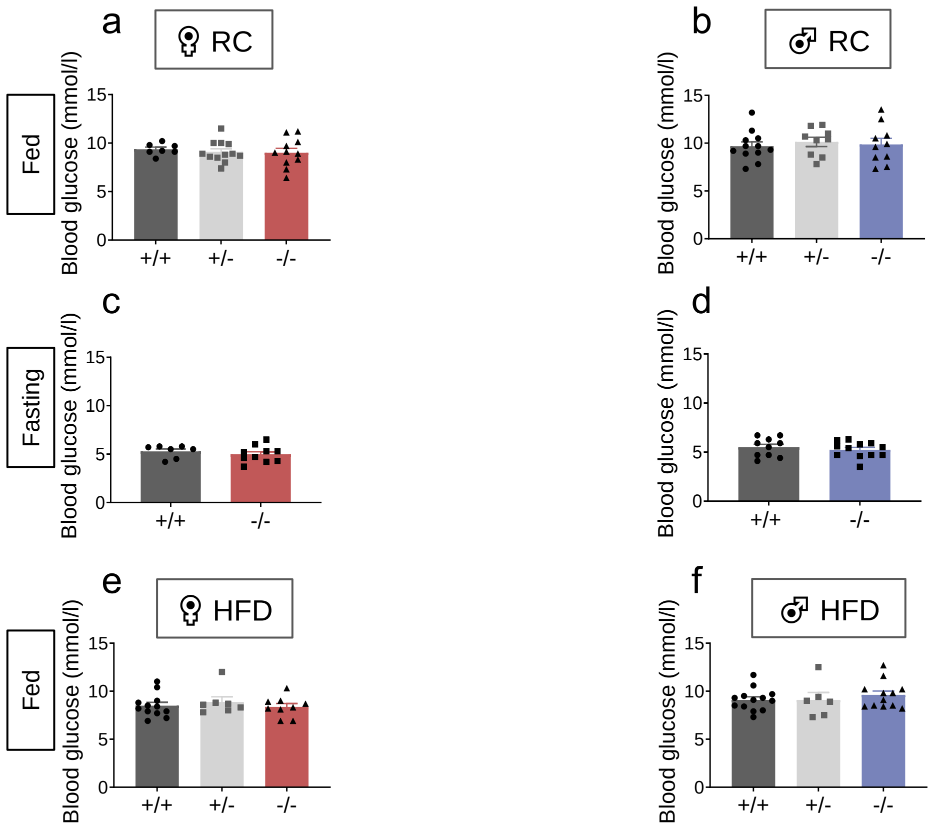

ESM Figure 5. *C2cd4b* mice glycemia. a-d. Fed and fasting glycemia in $C2cd4b^{+/+}$ and $C2cd4b^{-/-}$ mice on RC, at 18 and 20 weeks of age respectively (number of animals used for fed glycemia, $F^{+/+}$ n=7, $F^{+/-}$ n=12, $F^{-/-}$ n=11; $M^{+/+}$ n=12, $M^{+/-}$ n=9, $M^{-/-}$ n=10; fasting glycemia: $F^{+/+}$ n=7, $F^{-/-}$ n=10, $M^{+/+}$ n=10, $F^{-/-}$ n=12). e-f. Fasting glycemia in animals on HFD were measured at 23 weeks of age (number of animals used: $F^{+/+}$ n=9, $F^{-/-}$ n=5; $M^{+/+}$ n=5, $M^{-/-}$ n=7). Data were assessed for significance using an unpaired Student's t-test, or 2-way ANOVA where three genotypes were compared. Values represent means ± SEM.

ESM Fig. 6

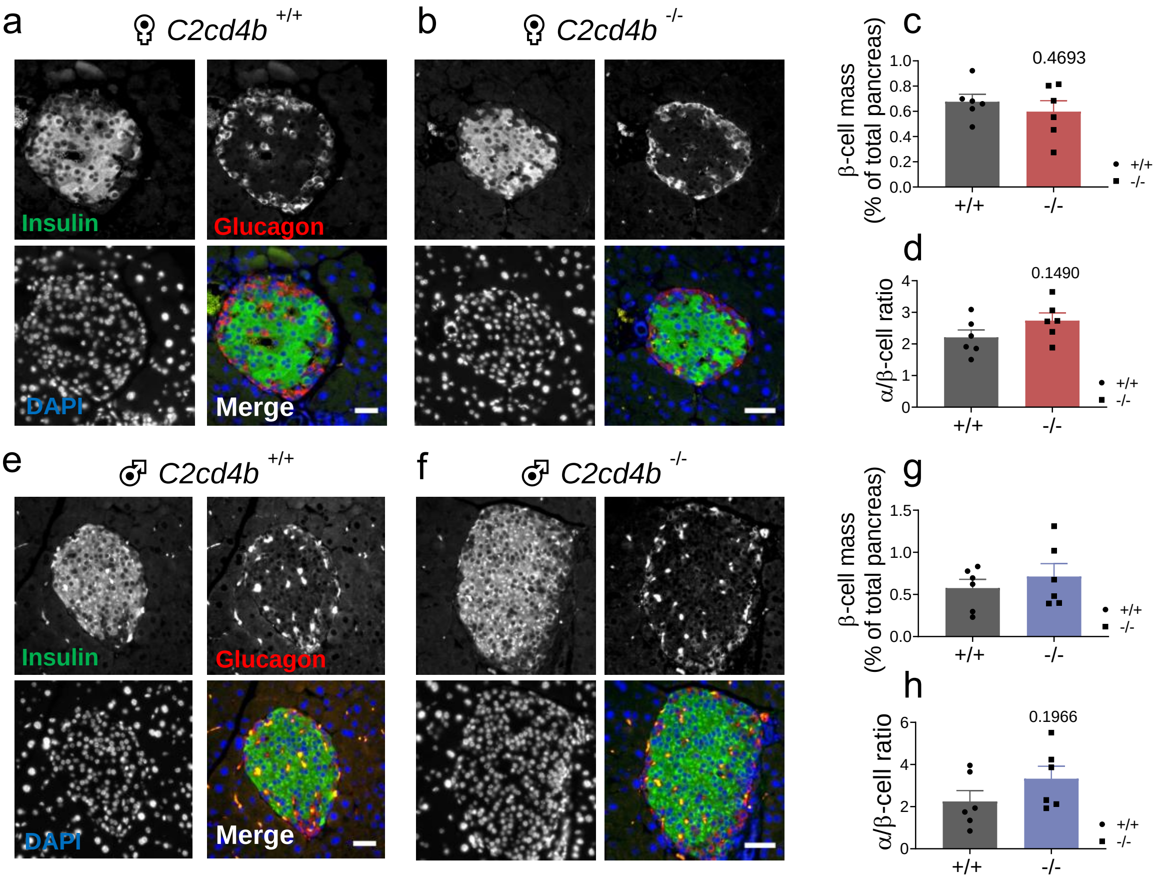

ESM Figure 6. β-cell mass in *C2cd4b* null mice. Data were collected from dissected pancreas from six mice per genotype at 24 weeks of age. For quantifications, three and two slides per female and male animals, respectively, were used. a,b,e,f. Immunohistochemistry was performed on slide sections with anti-insulin (shown in green), anti-glucagon (shown in red) antibodies, and DAPI (shown in blue). Scale bars= 30 μm. c,d,g,h. Area stained with insulin or glucagon antibodies were quantified to represent β-cell and α-cell mass, respectively, using ImageJ software. Data were assessed for significance using an unpaired Student’s t-test. Values represent mean ± SEM.

ESM Fig. 7

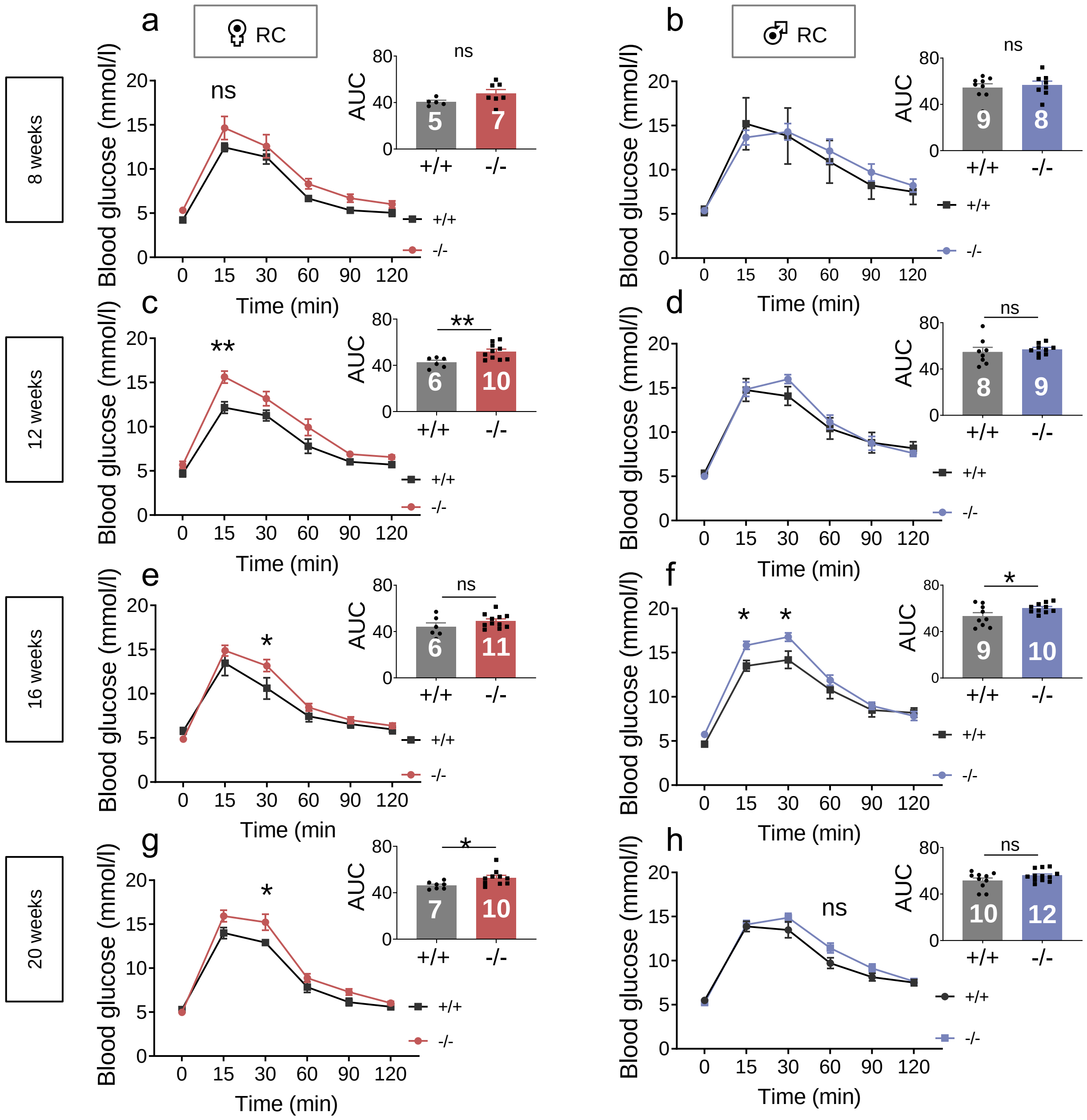

ESM Figure 7. Effect of deletion of *C2cd4b* on IPGTTs in mice maintained on RC. IPGTTs were performed on *C2cd4b* null male (b,d,f,h) and female (a,c,e,g) animals on RC at 8, 12, 16 and 20 weeks of age. *p<0.05, **p<0.01, ***p<0.001, data were assessed for significance using a 2-way ANOVA with Bonferroni's multiple comparison test. Inset: area under the curve (AUC) analysis; assessed for significance using an unpaired Student's t-test. Numbers in bar graphs represent the number of animals used (same number of samples used for glycemia and AUC (mmol/l x min) graphs). Values represent means ± SEM.

ESM Fig. 8

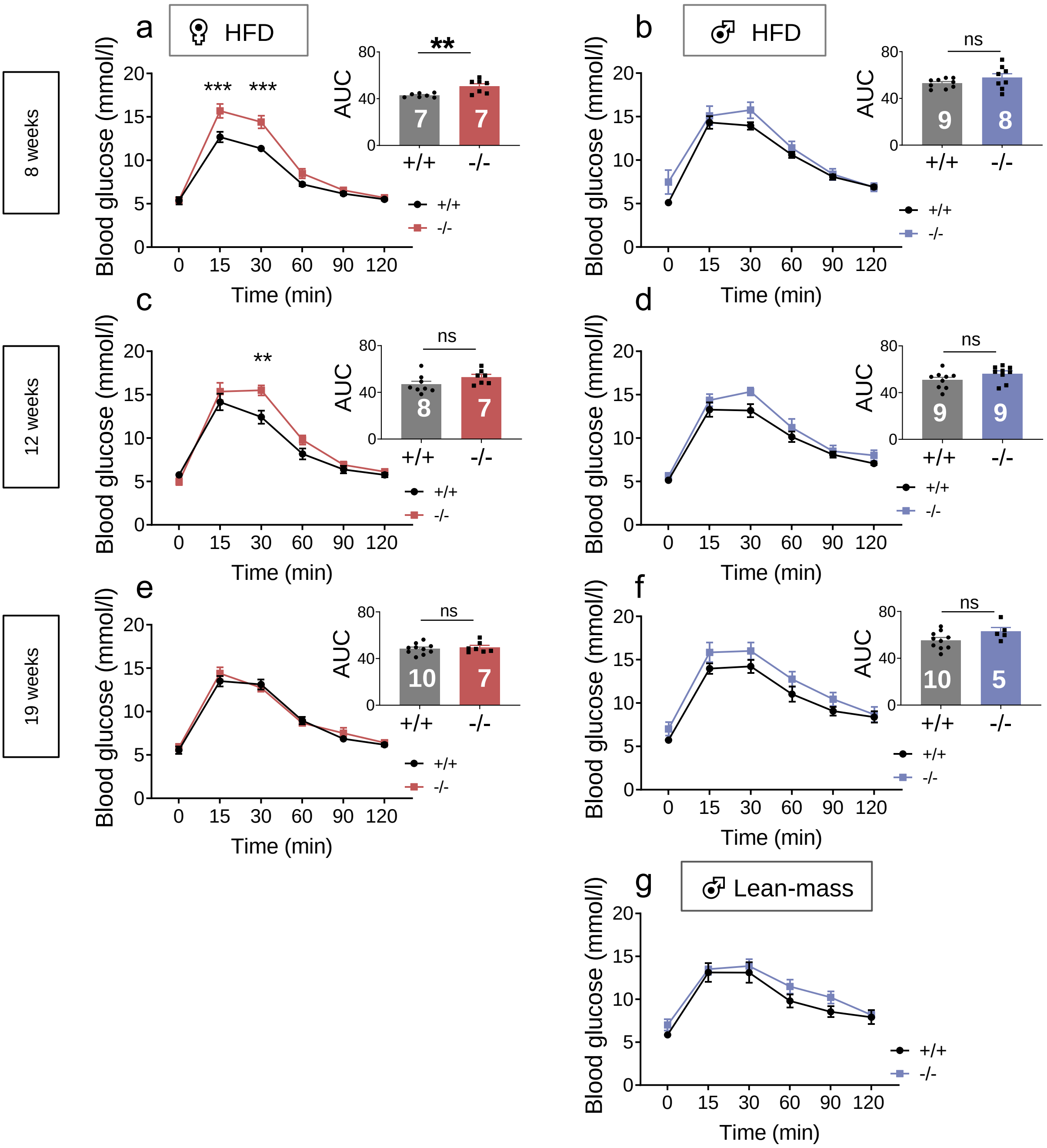

ESM Figure 8. Effect of deletion of *C2cd4b* on IPGTTs in animals maintained on HFD. a-f. IPGTTs were performed on *C2cd4b* null female (a,c,e) and male (b,d,f) animals on HFD at 8, 12, and 19 weeks of age. g. IPGTTs according to lean mass from *C2cd4b* male mice at 25 weeks of age. *p<0.05, **p<0.01, ***p<0.001, 2-way ANOVA with Bonferroni's multiple comparison test. Inset: area under the curve (AUC (mmol/l x min)) analysis; data were assessed for significance using an unpaired Student's t-test. Numbers in bar graphs represent the number of animals used (same number of samples used for Glycemia and AUC graphs). Values represent means ± SEM.

ESM Fig. 9

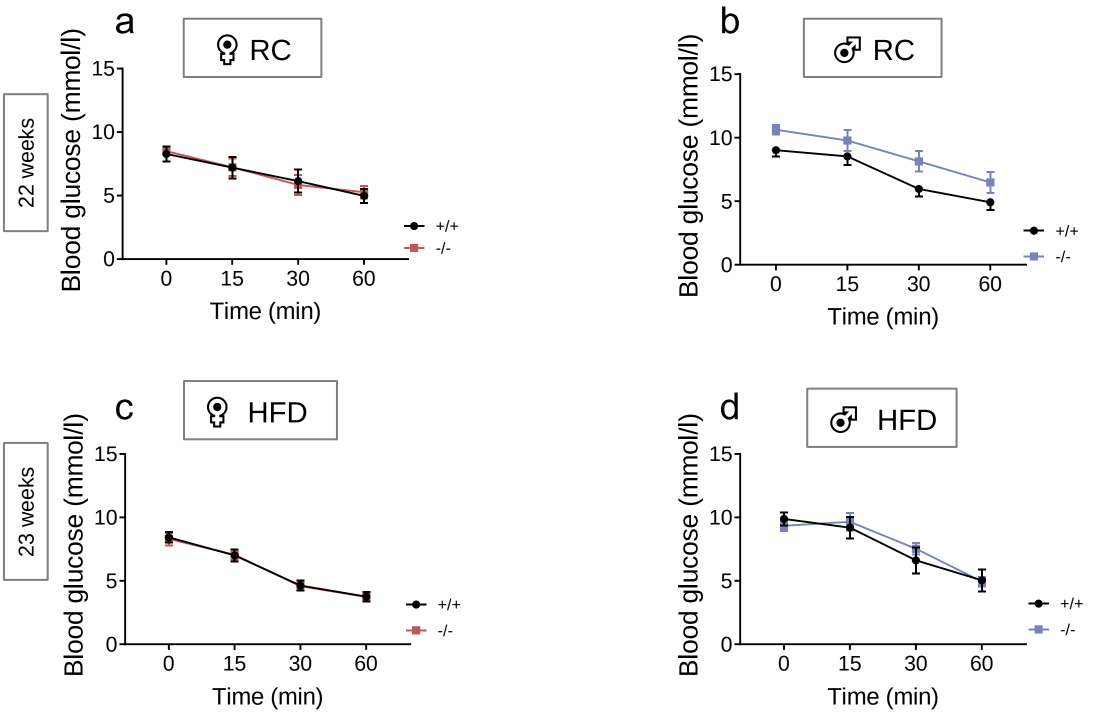

ESM Figure 9. Effect of deletion of *C2cd4b* on insulin sensitivity. a-b. *C2cd4b* null and WT male ($M^{+/+}$ n=9, $M^{-/-}$ n=13) and female ($F^{+/+}$ n=6, $F^{-/-}$ n=9) mice on RC were injected with a 1 or 0.75 Unit/kg body weight dose of insulin, respectively. c-d. HFD, *C2cd4b* null and WT male (n=8/genotype) and female ($F^{+/+}$ n=11, $F^{-/-}$ n=7) mice were injected with 1.5 or 0.75 Unit/kg body weight dose of insulin. Glycemia was monitored over a one-h period. Deletion of *C2cd4b* had no effect on insulin sensitivity in either sex, examined under RC or HFD. Data were assessed for significance using a 2-way ANOVA with Bonferroni's multiple comparison test. Values represent mean ± SEM.

ESM Fig. 10

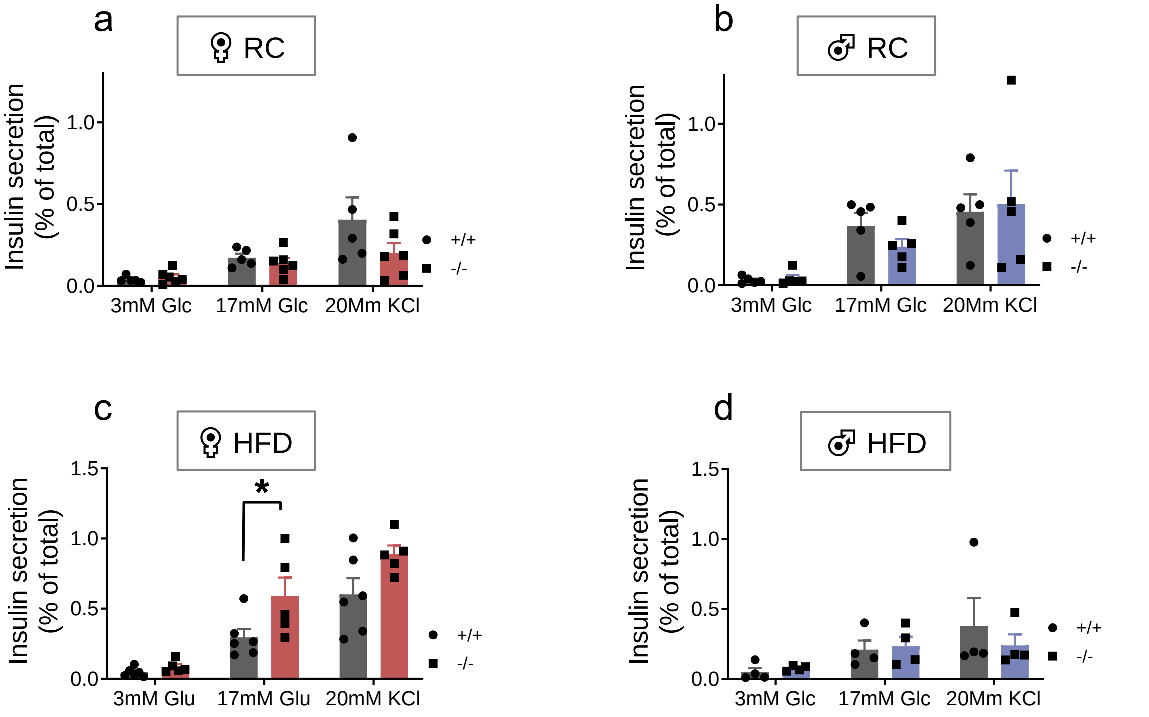

ESM Figure 10. Effect of deletion of *C2cd4b* on glucose or KCl-stimulated insulin secretion from isolated islets. Insulin secretion was measured in islets from *C2cd4b* mice maintained on RC (a-b) or HFD (c-d) at 24 weeks of age. (RC: $F^{+/+}$ n=5, $F^{-/-}$ n=6, M n=5/genotype; HFD: $F^{+/+}$ n=6 $F^{-/-}$ n=5, M n=4/genotype). * $p<0.05$, ** $p<0.01$, *** $p<0.001$, 2-way ANOVA with Bonferroni's multiple comparison test. Values represent mean ± SEM.

ESM Fig. 11

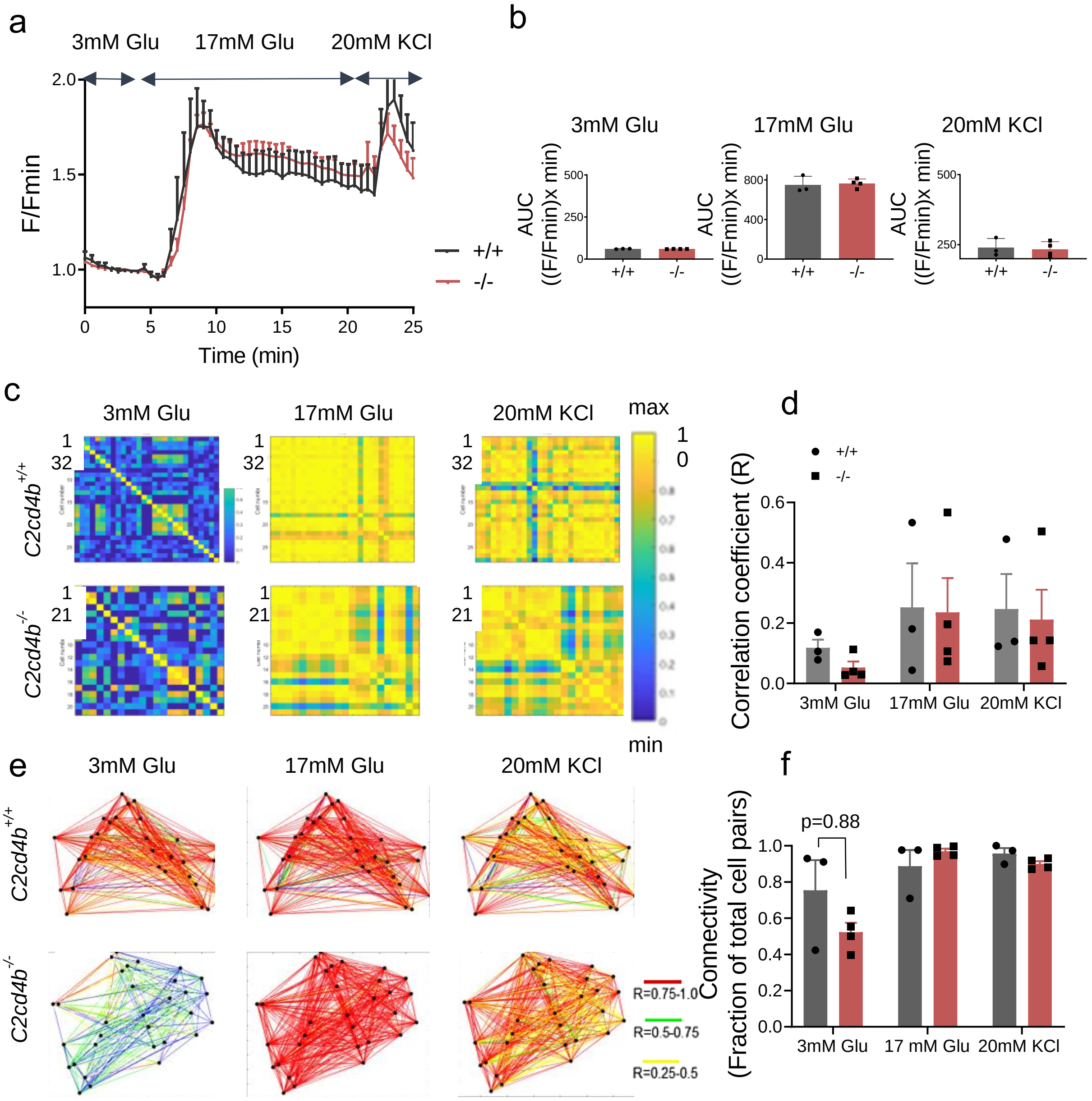

ESM Figure 11. Effect of deletion of *C2cd4b* on Ca^{2+} dynamics in isolated islets. Intracellular Ca^{2+} dynamics were assessed in isolated islets from females at 24 weeks of age maintained on RC, using spinning disk microscopy. a-b. No changes were observed in 17 mM glucose (Glu)- or 20 mM KCl-stimulated Ca^{2+} dynamics between *C2cd4b* null and WT ($F^{+/+}$ n=3 and $F^{-/-}$ n=4 per experiments, two acquisitions were performed in each experiment and between 8-12 islets were assessed in each acquisition; assessed for significance using an unpaired Student's t-test). c-f. No significant changes in β-cell connectivity and in the correlation coefficient was observed between the null and WT islets (three islets assessed in duplicate for each genotype; data were assessed for significance using a 2-way ANOVA with Bonferroni's multiple comparison test. Values represent mean ± SEM.

ESM Fig. 12

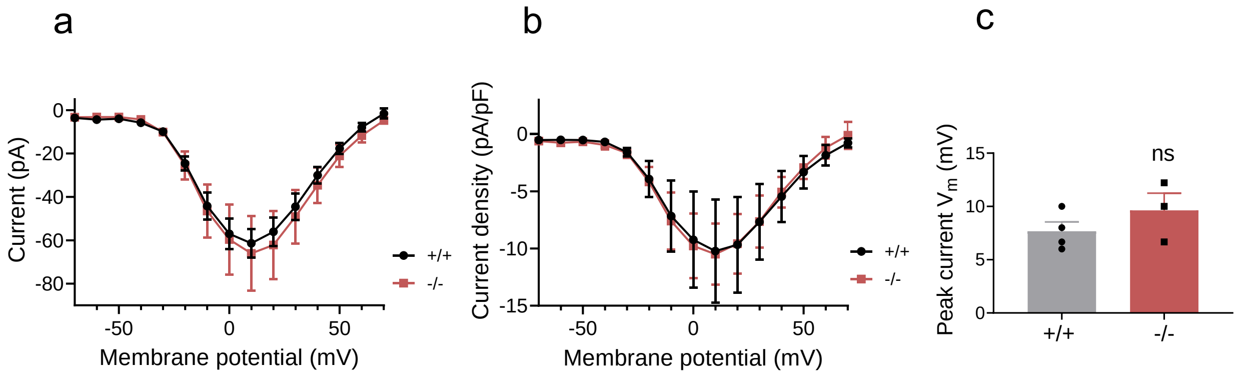

ESM Figure 12. *C2cd4b* deletion does not affect β cell voltage-dependent calcium channel activity. a. Average VDCC currents recorded from control *C2cd4b*$^{+/+}$ and *C2cd4b*$^{-/-}$ β cells in response to 10 mV steps from −70 to 70 mV (*C2cd4b*$^{+/+}$, n=18 β-cells (from 4 mice); *C2cd4b*$^{-/-}$, n=26 cells (from 3 mice). b. Average WT and *C2cd4b* null β cell VDCC currents normalized to cell capacitance. c. average peak WT (gray) and *C2cd4b* null (green) β cell VDCC currents normalised to cell capacitance. Data were assessed for significance using an unpaired Student's t-test. Values represent mean ± SEM.

.

ESM Fig. 13

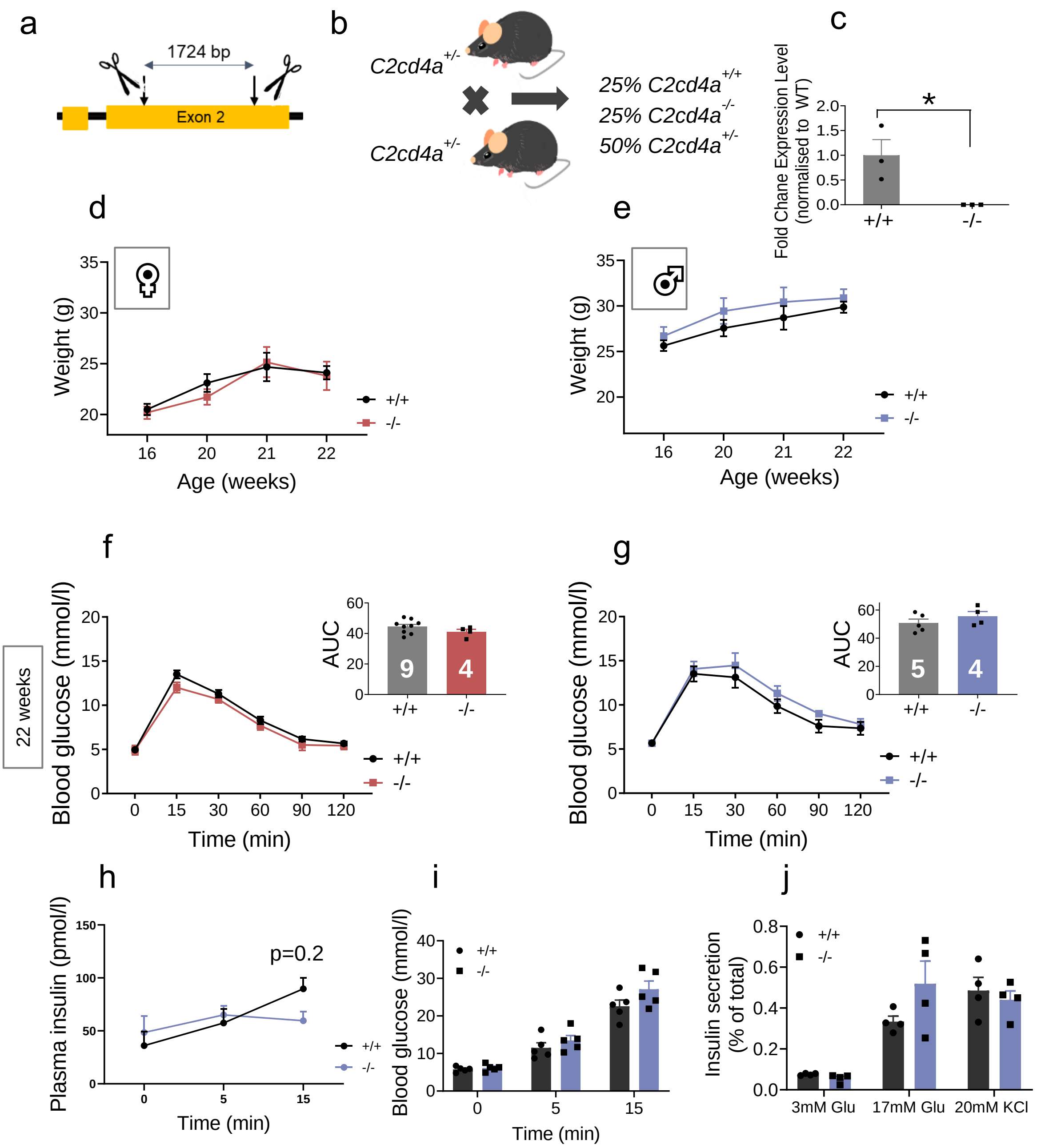

ESM Figure 13. *C2cd4a* null mice. a. *C2cd4a* mouse strain was generated by the IMPC using CRISPR/Cas9 to target 1724 bps from exon 2 of *C2cd4a* gene (See Methods). b. Inter-crossing of heterozygous mice resulted in the generation of wild type (WT, *C2cd4a*$^{+/+}$), heterozygous (*C2cd4a*$^{+/-}$) and null (*C2cd4a*$^{-/-}$) littermates. c. RT-q-PCR on RNA in isolated islets revealed a significant decrease in *C2cd4a* mRNA levels in homozygous animals (p=0.034), (n=3/genotype). *p<0.05, data were assessed for significance using an unpaired Student's t-test. d,e. Body weight of *C2cd4a* females (d) and males (e) at 16, 20. 21 and 22 weeks of age (F$^{+/+}$ n=4-11, F$^{-/-}$ n=4-9, M$^{+/+}$ n=2-8, M$^{-/-}$ n=3-6 animals per stage). Mixed-effect analysis, Tukey's multiple comparison test. f,g. IPGTTs were performed on *C2cd4b* female (f) and male (g) mice maintained on RC at 22 weeks of age. Inset: area under the curve (AUC (mmol/l x min)) analysis, assessed for significance using an unpaired Student's t-test (same number of samples used for glycemia and AUC graphs). h,i. *In vivo* glucose-stimulated insulin secretion were performed on *C2cd4a* null male animals maintained on RC at 21 weeks of age. Blood samples were collected, and plasma insulin levels and glycemia measured at 5 and 15 min. after injection of glucose at 3 g/kg body weight. n=5/genotype. j. *In vitro* insulin levels in *C2cd4a* null *male* and WT mice maintained on RC. Measurements of insulin secretion were performed after stimulating isolated islets with 17 mM glucose or 20 mM KCl (n= 4/genotype). . *p<0.05, **p<0.01, ***p<0.001, data were assessed for significance using a 2-way ANOVA with Bonferroni's multiple comparison test. Values represent mean ± SEM.

ESM Fig. 14

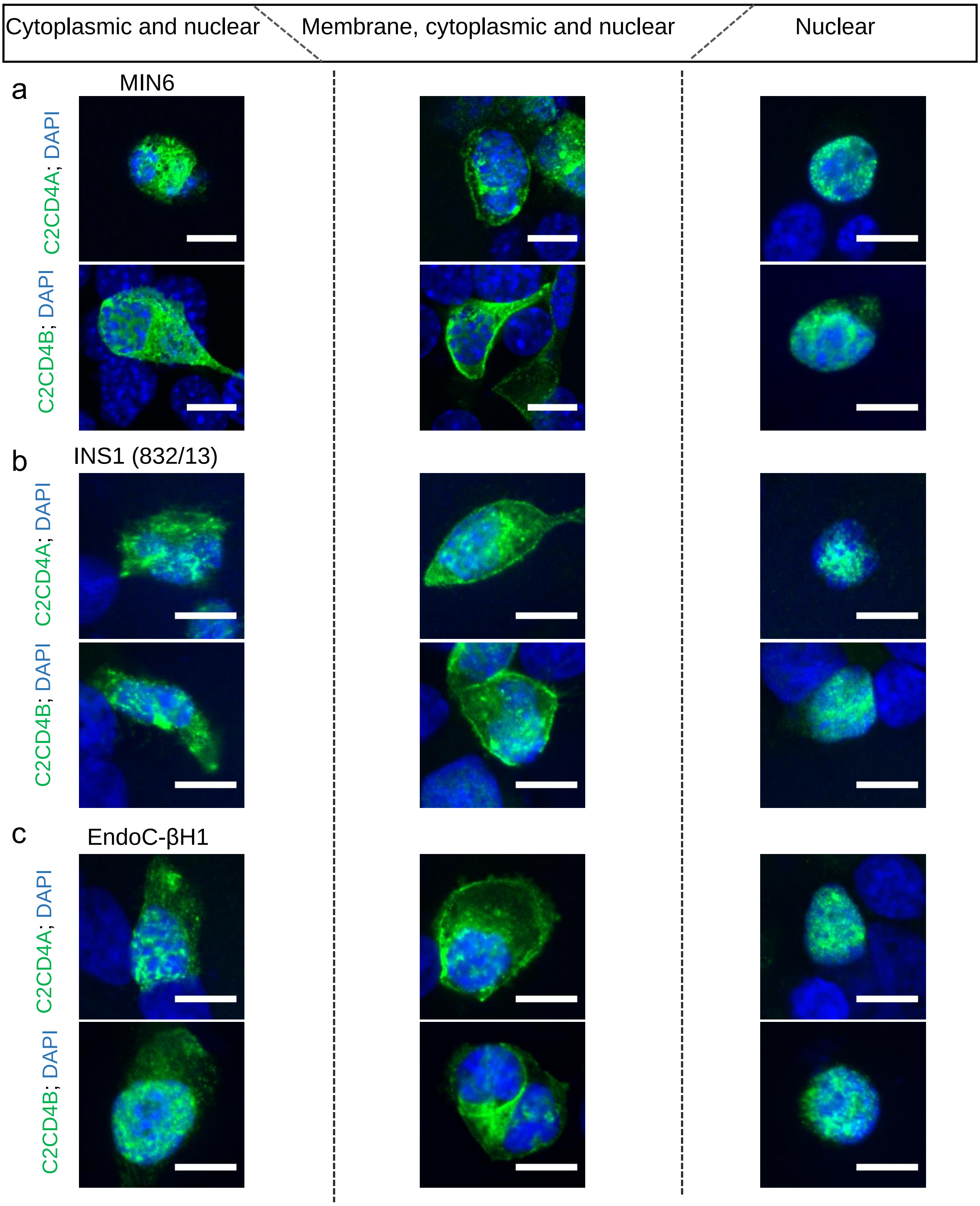

ESM Figure 14. Sub-cellular localisation of C2CD4A and C2CD4B in β-cells. Cells were transfected with either C2CD4A-FLAG or C2CD4B-FLAG tagged constructs and immunohistochemistry was performed using anti-FLAG (shown in green) antibody. Three main localisation patterns were observed by visualising C2CD4A (green) and C2CD4B (green) proteins in (a) MIN6, (b) INS1 (832/13) and (c) EndoC βH1 cells. DAPI is shown in blue. Scale bars=10 μm.

ESM Fig. 15

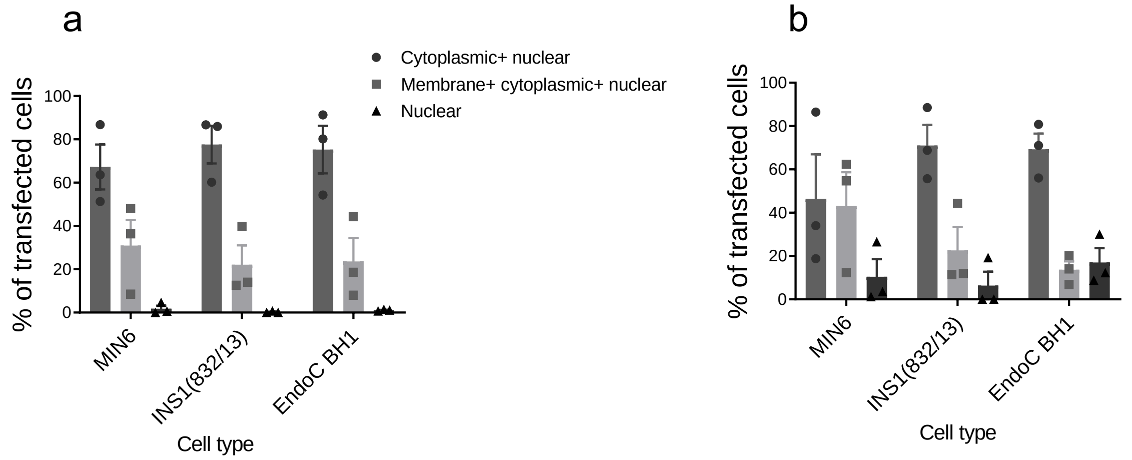

ESM Figure 15. Quantification of different localisation patterns in different β-cell types using C2CD4A: FLAG (a) and C2CD4B: FLAG-tagged (b) constructs. The quantifications were performed in three independent experiments. In each case, two separate cultures were analysed. Approximately 150 cells from two separate cultures were analysed in each experiment. No changes were detected in the proportions of the different localisation patterns between different cell types. Data were assessed for significance using a 2-way ANOVA with Bonferroni's multiple comparison test. Values represent means ± SEM.

ESM Fig. 16

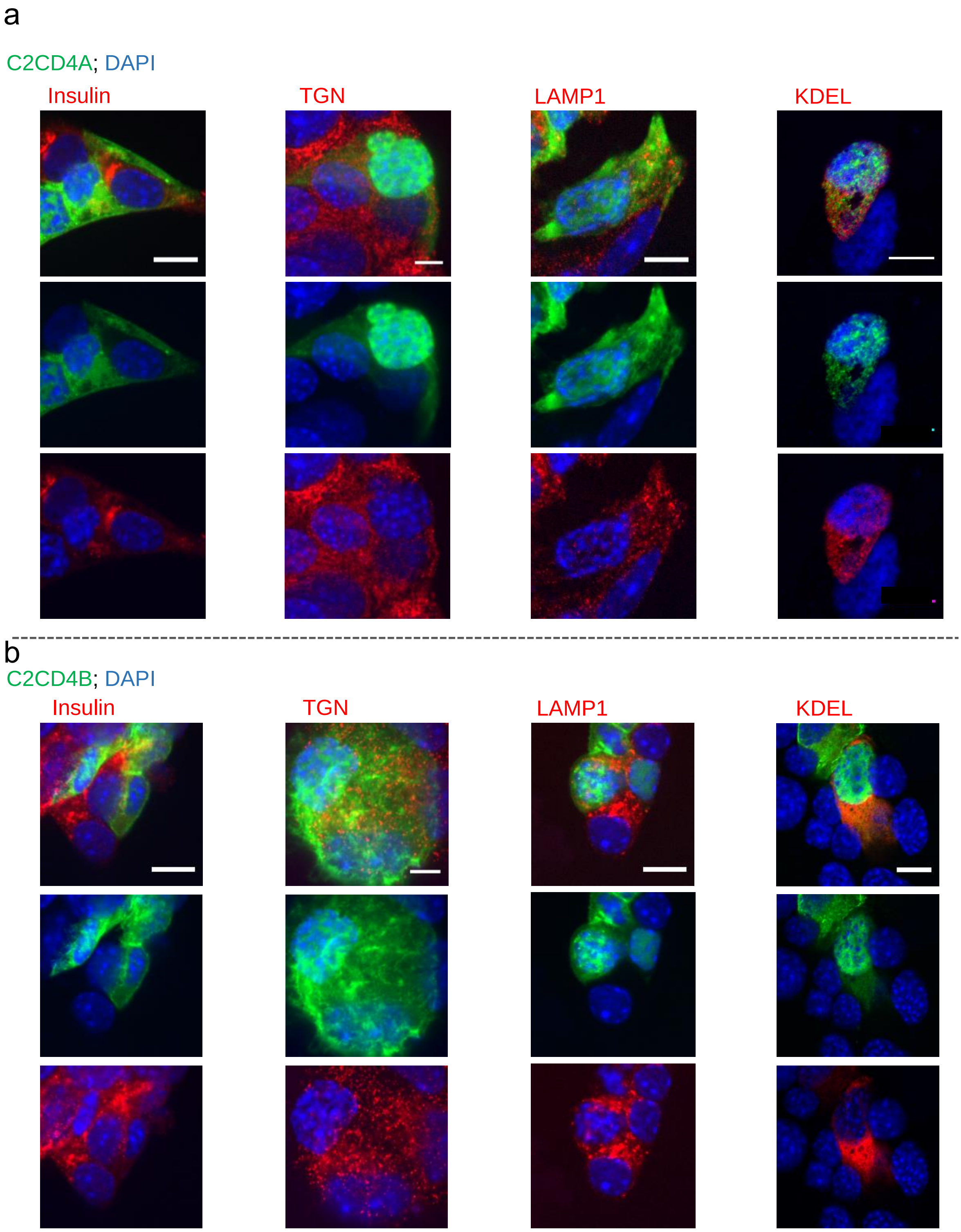

ESM Figure 16. Sub-cellular co-localisation of C2CD4A and C2CD4B in MIN6 cells. a. Sub-cellular co-localisation of C2CD4A (in green) with insulin, TGN (TGN46), lysosomes (LAMP1) and ER (KDEL) (all in red). b. Sub-cellular co-localisation of C2CD4B (in green) with insulin, TGN, lysosomes (LAMP1) and ER (KDEL) (all in red). DAPI staining shown in blue. Scale bars= 10 µm.

References

1. Owen BM, Bookout AL, Ding X, Lin VY, Atkin SD, Gautron L, et al. FGF21 contributes to neuroendocrine control of female reproduction. Nat Med [Internet]. 2013;19(9):1153–6. Available from: https://doi.org/10.1038/nm.3250
2. Ravier MA, Rutter GA. Isolation and Culture of Mouse Pancreatic Islets for Ex Vivo Imaging Studies with Trappable or Recombinant Fluorescent Probes. In: Ward A, Tosh D, editors. Mouse Cell Culture: Methods and Protocols [Internet]. Totowa, NJ: Humana Press; 2010. p. 171–84. Available from: https://doi.org/10.1007/978-1-59745-019-5_12
3. Nguyen-Tu M-S, da Silva Xavier G, Leclerc I, Rutter GA. Transcription factor-7-like 2 (TCF7L2) gene acts downstream of the Lkb1/Stk11 kinase to control mTOR signaling, β cell growth, and insulin secretion. J Biol Chem [Internet]. 2018/07/02. 2018 Sep 7;293(36):14178–89. Available from: https://pubmed.ncbi.nlm.nih.gov/29967064
4. Westerfield M. The Zebrafish Book. A Guide for the Laboratory Use of Zebrafish (Danio rerio), 3rd Edition. Eugene, OR, Univ Oregon Press. 1995;
5. Fisher S, Grice EA, Vinton RM, Bessling SL, Urasaki A, Kawakami K, et al. Evaluating the biological relevance of putative enhancers using Tol2 transposon-mediated transgenesis in zebrafish. Nat Protoc. 2006;1(3):1297–305.
6. Fisher S, Grice EA, Vinton RM, Bessling SL, McCallion AS. Conservation of RET regulatory function from human to zebrafish without sequence similarity. Science (80-). 2006;312(5771):276–9.
7. Flasse LC, Pirson JL, Stern DG, Von Berg V, Manfroid I, Peers B, et al. Ascl1b and Neurod1, instead of Neurog3, control pancreatic endocrine cell fate in zebrafish. BMC Biol. 2013;11(1):78.
8. Mavropoulos A, Devos N, Biemar F, Zecchin E, Argenton F, Edlund H, et al. sox4b is a key player of pancreatic alpha cell differentiation in zebrafish. Dev Biol. 2005 Sep;285(1):211–23.
9. Thisse C, Thisse B. High-resolution in situ hybridization to whole-mount zebrafish embryos. Nat Protoc. 2008;3(1):59–69.
10. Salem V, Silva LD, Suba K, Georgiadou E, Neda Mousavy Gharavy S, Akhtar N, et al. Leader β-cells coordinate Ca2+ dynamics across pancreatic islets in vivo. Nat Metab [Internet]. 2019;1(6):615–29. Available from: https://doi.org/10.1038/s42255-019-

0075-2

11. Hodson DJ, Mitchell RK, Bellomo EA, Sun G, Vinet L, Meda P, et al. Lipotoxicity disrupts incretin-regulated human β cell connectivity. J Clin Invest. 2013;123(10):4182–94.

12. Patro R, Duggal G, Love MI, Irizarry RA, Kingsford C. Salmon provides fast and bias-aware quantification of transcript expression. Nat Methods [Internet]. 2017 Mar 6;14:417. Available from: https://doi.org/10.1038/nmeth.4197

13. Love MI, Huber W, Anders S. Moderated estimation of fold change and dispersion for RNA-seq data with DESeq2. Genome Biol [Internet]. 2014;15(12):550. Available from: https://doi.org/10.1186/s13059-014-0550-8

14. Anders S, McCarthy DJ, Chen Y, Okoniewski M, Smyth GK, Huber W, et al. Count-based differential expression analysis of RNA sequencing data using R and Bioconductor. Nat Protoc [Internet]. 2013 Aug 22;8:1765. Available from: https://doi.org/10.1038/nprot.2013.099

15. Miyazaki J-I, Araki K, Yamato E, Ikegami H, Asano T, Shibasaki Y, et al. Establishment of a Pancreatic β Cell Line That Retains Glucose-Inducible Insulin Secretion: Special Reference to Expression of Glucose Transporter Isoforms*. Endocrinology [Internet]. 1990;127(1):126–32. Available from: https://doi.org/10.1210/endo-127-1-126

16. Millership SJ, Da Silva Xavier G, Choudhury AI, Bertazzo S, Chabosseau P, Pedroni SM, et al. Neuronatin regulates pancreatic β cell insulin content and secretion. J Clin Invest [Internet]. 2018/07/09. 2018 Aug 1;128(8):3369–81. Available from: https://www.ncbi.nlm.nih.gov/pubmed/29864031

17. Cox J, Hein MY, Luber CA, Paron I, Nagaraj N, Mann M. Accurate Proteome-wide Label-free Quantification by Delayed Normalization and Maximal Peptide Ratio Extraction, Termed MaxLFQ. Mol & Cell Proteomics [Internet]. 2014 Sep 1;13(9):2513 LP – 2526. Available from: http://www.mcponline.org/content/13/9/2513.abstract

18. Perez-Riverol Y, Csordas A, Bai J, Bernal-Llinares M, Hewapathirana S, Kundu DJ, et al. The PRIDE database and related tools and resources in 2019: improving support for quantification data. Nucleic Acids Res. 2019 Jan;47(D1):D442–50.

19. Benner C, van der Meulen T, Caceres E, Tigyi K, Donaldson CJ, Huising MO. The transcriptional landscape of mouse beta cells compared to human beta cells reveals notable species differences in long non-coding RNA and protein-coding gene

expression. BMC Genomics. 2014;15(1).

20. Kone M, Pullen TJ, Sun G, Ibberson M, Martinez-Sanchez A, Sayers S, et al. LKB1 and AMPK differentially regulate pancreatic β-cell identity. FASEB J. 2014;28(11):4972–85.

21. Blodgett DM, Nowosielska A, Afik S, Pechhold S, Cura AJ, Kennedy NJ, et al. Novel observations from next-generation RNA sequencing of highly purified human adult and fetal islet cell subsets. Diabetes. 2015;64(9):3172–81.

22. Marullo L, El-Sayed Moustafa JS, Prokopenko I. Insights into the genetic susceptibility to type 2 diabetes from genome-wide association studies of glycaemic traits. Curr Diab Rep. 2014;14(11):1–30.

23. Kubosaki A, Nakamura S, Clark A, Morris JF, Notkins AL. Disruption of the transmembrane dense core vesicle proteins IA-2 and IA-2β causes female infertility. Endocrinology. 2006;147(2):811–5.

24. Saeki K, Zhu M, Kubosaki A, Xie J, Lan MS, Notkins AL. Targeted disruption of the protein tyrosine phosphatase-like molecule IA-2 results in alterations in glucose tolerance tests and insulin secretion. Diabetes. 2002;51(6):1842–50.

25. Cruciani-Guglielmacci C, Bellini L, Denom J, et al (2017) Molecular phenotyping of multiple mouse strains under metabolic challenge uncovers a role for Elovl2 in glucose-induced insulin secretion. Mol Metab 6(4):340–351.
